# Supplementary material for: Polygenic risk scores for the prediction of common cancers in East Asians: A population-based prospective cohort study
Source: eLife. 2023 Mar 27;12:e82608. doi: 10.7554/eLife.82608 (PMC10159619; doi:10.7554/eLife.82608)
Supplement: Source code 1. [file elife-82608-code1.zip › Sourcecode1.docx]

Polygenic risk scores for the prediction of common cancers in East Asians: A population-based prospective cohort study

hopj and lijm

2/24/2022

Note: The codes here uses Linux system and Windows/ Mac OS.

# Download and compute PRS scores

(Linux system)

#####################################################
# DOWNLOAD PRS
# ONLY SCORES COMPRISING <100,000 SNPS DOWNLOADED
#####################################################

cd /mnt/projects/lijm1/humgen7/schs-pgs/

## START download.sh
curl $1 -o $2
gunzip $2.txt.gz
## END download.sh

R
data <- read.csv("pgs_all_metadata.txt",header=T,sep="\t",stringsAsFactor=F)
data$Number.of.Variants <- as.numeric(as.character(gsub(",","",data$Number.of.Variants)))
data <- data[data$Number.of.Variants<100000,]
out <- paste0("./download.sh ", data$FTP.link," ",data$Polygenic.Score..PGS..ID,".txt.gz")
write.table(out, "temp", row.names=F,col.names=F,quote=F,sep=" ")

chmod 755 temp
./temp

rm temp


###########################
# CREATE FOLDER STRUCTURE
###########################

ls P* | sed s/".txt"/""/g > temp
awk '{print "mkdir " $1 }' temp> temp2
chmod 755 temp2
./temp2


ls PGS*.txt > temp3
paste temp3 temp > temp4
awk '{print "mv " $1 " " $2}' temp4 > temp5
chmod 755 temp5
./temp5

rm temp*

###########################
# CREATE SIEVE FILES
###########################


#### BEGIN preprocess.R

d <- read.table("weight",header=T,sep="\t")

if(is.na(match("rsID",colnames(d)))==F&is.na(match("chr_position",colnames(d)))==F){
a <- as.data.frame(as.character(d$rsID))
b <- as.data.frame(as.character(d$chr_position))
colnames(b) <- colnames(a)
c <- rbind(a,b)
write.table(c,"sieve",row.names=F,col.names=F,quote=F,sep="\t")
}

if(is.na(match("rsID",colnames(d)))==F&is.na(match("chr_position",colnames(d)))==T){
a <- as.data.frame(as.character(d$rsID))
write.table(a,"sieve",row.names=F,col.names=F,quote=F,sep="\t")
}

if(is.na(match("rsID",colnames(d)))==T&is.na(match("chr_position",colnames(d)))==F){
b <- as.data.frame(as.character(d$chr_position))
write.table(b,"sieve",row.names=F,col.names=F,quote=F,sep="\t")
}

#### END preprocess.R


## BEGIN preprocess.sh
cd $1
grep -v "#" P* > weight
R --no-save < ../preprocess.R
## END preprocess.sh


ls -d PGS* > temp
awk '{print "./preprocess.sh " $1 }' temp > temp2
chmod 755 temp2
./temp2
cat P*/sieve > sieve
rm temp*

###########################
# EXTRACT VARIANTS
###########################

## CHECK AVAILABLE VARIANT MATCH
autorun "grep -wFf sieve /mnt/projects/dorajoor/cobseq/SCHS/21828/impute/info/info-%-all | cut -f2 -d ' ' > sieve-%" --enum 1-22


###########################
# CREATE PLINK FILE
###########################

## START extract.sh
plink1.9 --gen /mnt/projects/dorajoor/cobseq/SCHS/21828/impute/out/out-$1-all.gz --sample schs.sample --oxford-single-chr $1 --out temp-chr$1 --threads 99
plink1.9 --bfile temp-chr$1 --extract sieve-$1 --make-bed --out schs-chr$1
rm temp-chr$1.*
## END extract.sh

autorun "qsub -pe OpenMP 8 -cwd -l h_rt=24:00:00,mem_free=100G -b y ./extract.sh %" --enum 1-22 -t

ls schs-chr*.bim | sed s/".bim"/""/g > mergelist

plink1.9 --merge-list mergelist --out schs --threads 99
rm mergelist sieve-*


###########################
## CALCULATE PRS
###########################

## BEGIN run.sh
cd $1
R --no-save < /mnt/projects/lijm1/humgen7/schs-pgs/Rscript
plink1.9 --bfile /mnt/projects/lijm1/humgen7/schs-pgs/schs --score /mnt/projects/lijm1/humgen7/schs-pgs/$1/score 1 2 3 --out /mnt/projects/lijm1/humgen7/schs-pgs/$1
plink1.9 --bfile /mnt/projects/lijm1/humgen7/schs-pgs/schs --score /mnt/projects/lijm1/humgen7/schs-pgs/$1/score 1 2 3 sum --out /mnt/projects/lijm1/humgen7/schs-pgs/$1-sum
rm ../$1.nosex
rm ../$1-sum.nosex
## END run.sh

## CREATE BASE BIM FILE
R
b <- read.table("../schs.bim",header=F,stringsAsFactors=F)
b$rsid <- gsub("\\:.*","",b$V2)
b$rsid <- gsub(".*-", "", b$rsid)
b$rsid[nchar(as.character(b$rsid))<2] <- NA
save.image("schs.Rdata")


## BEGIN Rscript

R

load("../schs.Rdata")

w <- read.table("weight",header=T,sep="\t",stringsAsFactors=F)
w <- w[,colnames(w)%in%c("rsID","chr_name","chr_position","effect_allele","effect_weight")]
w$effect_allele[w$effect_allele==""] <- "-"
head(b)
head(w)

if (is.na(match("rsID",colnames(w)))==F) {
out1 <- merge(b,w,by.x=c("rsid"),by.y=c("rsID"),all.y=T)
out1$V2[is.na(out1$V2)==T] <- out1$rsid[is.na(out1$V2)==T]
out1 <- out1[,c("V1","V2","V4","V5","V6","effect_allele","effect_weight")]
miss1 <- length(out1$V6[is.na(out1$V6)==T])
print(miss1/nrow(w))
}

if (is.na(match("chr_position",colnames(w)))==F) {
out2 <- merge(b,w,by.x=c("V1","V4"),by.y=c("chr_name","chr_position"),all.y=T)
out2 <- out2[,c("V1","V2","V4","V5","V6","effect_allele","effect_weight")]
miss2 <- length(out2$V6[is.na(out2$V6)==T])
print(miss2/nrow(w))
}

if (is.na(match("rsID",colnames(w)))==T) {
out <- out2[,c("V2","effect_allele","effect_weight")]
write.table(out,"score",row.names=F,col.names=F,quote=F,sep="\t")}

if (is.na(match("chr_position",colnames(w)))==T) {
out <- out1[,c("V2","effect_allele","effect_weight")]
write.table(out,"score",row.names=F,col.names=F,quote=F,sep="\t")}

if(is.na(match("rsID",colnames(w)))==F&is.na(match("chr_position",colnames(w)))==F){

if(miss1>miss2){
out <- out2[,c("V2","effect_allele","effect_weight")]
write.table(out,"score",row.names=F,col.names=F,quote=F,sep="\t")}

if(miss2>miss1){
out <- out1[,c("V2","effect_allele","effect_weight")]
write.table(out,"score",row.names=F,col.names=F,quote=F,sep="\t")}

if(miss1==miss2){
out <- out2[,c("V2","effect_allele","effect_weight")]
write.table(out,"score",row.names=F,col.names=F,quote=F,sep="\t")}

}

## END Rscript


awk '{print "./run.sh " $1 }' prs | split -l 100 -d
chmod 755 x*
autorun "qsub -pe OpenMP 8 -cwd -l h_rt=24:00:00,mem_free=30G -b y ./x%" --enum 00-17 -t


#####################
# CHECKS
#####################

grep valid *-sum.log | cut -f1,2 -d " " | cut -f1 -d "-" > temp1
grep valid *-sum.log | cut -f2 -d " " > temp2
paste temp1 temp2 > temp
rm temp1 temp2

# PREPARING ANALYTICAL DATASET

(eligibility criteria) (Windows/Mac OS)

## Get profiles to merge

cd "R:/HG/HG7/Private/Datasets/SCHS From Kar Seng/projects/2022-common_cancers"
ls "R:/HG/HG7/Private/Datasets/PGS Catalog/PGS/schs-pgs/"*.profile > data/schs-pgs.list.of.profiles

cd "R:/HG/HG7/Private/Datasets/PGS Catalog/PGS/schs-pgs"
ls "R:/HG/HG7/Private/Datasets/PGS Catalog/PGS/schs-pgs/"*.profile > "R:/HG/HG7/Private/Datasets/SCHS From Kar Seng/projects/2022-common_cancers/data/schs-pgs.list.of.profiles"


cd "/Volumes/research/HG/HG7/Private/Datasets/PGS Catalog/PGS/schs-pgs"
ls *sum.profile > "/Volumes/research/HG/HG7/Private/Datasets/SCHS From Kar Seng/projects/2022-common_cancers/data/schs-pgs.list.of.profiles"

## Selection of PGS - common cancers

# setwd("R:/HG/HG7/Private/Datasets")
setwd("/Volumes/research/HG/HG7/Private/Datasets")
library(stringr)
common <- read.csv("SCHS From Kar Seng/projects/2022-common_cancers/data/PGS_proportion_of_variants_missing.csv")
PATH <- getwd()
pgs.list <- read.table("SCHS From Kar Seng/projects/2022-common_cancers/data/schs-pgs.list.of.profiles",sep="\t")
pgs.list <- cbind(pgs.list,pgs.list)
pgs.list[,2] <- str_replace(pgs.list[,2],"R:/HG/HG7/Private/Datasets/PGS Catalog/PGS/schs-pgs/","")
pgs.list[,2] <- str_replace(pgs.list[,2],"-sum.profile","")
pgs.list[,1] <- paste0(PATH,"/PGS Catalog/PGS/schs-pgs/",pgs.list[,1])
pgs.list <- pgs.list[pgs.list[,2]%in%as.character(common$PGS.ID),]

write.table(pgs.list,"SCHS From Kar Seng/projects/2022-common_cancers/data/common.pgs.list.scoresum",row.names = F,col.names = F,quote = F,sep="\t")

## Adding common cancers PRS to data

# setwd("R:/HG/HG7/Private/Datasets/SCHS From Kar Seng/projects/2022-common_cancers")
setwd("/Volumes/research/HG/HG7/Private/Datasets/SCHS From Kar Seng/projects/2022-common_cancers")

pheno <- read.csv("data/original_data/Telomere_v4_datasets.csv")
colnames(pheno)[str_detect(colnames(pheno),"FID")] <- "FID"

list.profiles <- read.table("data/common.pgs.list.scoresum",sep="\t")
colnames(list.profiles) <- c("path","name")

PRO = list.profiles$name[1]

out <- read.table(as.character(list.profiles$path[list.profiles$name==PRO]),header=T)
colnames(out)[4:6] <- paste0(colnames(out)[4:6],"_",PRO)
out <- out[,c(1:3,6)]

i=0
for(PRO in list.profiles$name[-1]){
 temp <- read.table(as.character(list.profiles$path[list.profiles$name==PRO]),header=T)
 colnames(temp)[4:6] <- paste0(colnames(temp)[4:6],"_",PRO)
 out <- merge(out,temp[,c(1,2,6)],by=c("FID","IID"),all=T)
 print(i)
 i=i+1
}

output <- merge(pheno,out,by=c("FID","IID"),all=T)

dim(output)

write.table(output,"data/data_SCHS_nn25759_common_scoresum_22020906.txt",quote=F,row.names=F,sep=",")
# rm(list=ls())

# setwd("R:/HG/HG7/Private/Datasets/SCHS From Kar Seng/projects/2022-common_cancers")
setwd("/Volumes/research/HG/HG7/Private/Datasets/SCHS From Kar Seng/projects/2022-common_cancers")
library(stringr)

data0 <- read.table("data/data_SCHS_nn25759_common_scoresum_22020906.txt",header=T,sep=",")

data <- data0[!is.na(data0[,which(str_starts(colnames(data0),"SCORESUM"))[1]]), ] #21828

colnames(data)[colnames(data)=="V9"] <- "V9_gender"
missing <- data[is.na(data$D1),] # information on individuals with Cancer diagnosis before recruitment/ missing cancer was not provided status
dim(missing)
write.csv(missing,"data/missing_n134.csv",row.names = F)

data <- data[!is.na(data$D1),] #21694
colnames(data)[colnames(data)=="D1"] <- "age_recruitment"


data$PHENO.Breast <- data$breast
table(data$breast)

data$PHENO.Colorectal <- data$crc
table(data$crc)

data$PHENO.Lung <- data$lung
table(data$lung)

data$PHENO.Prostate <- data$prostate
table(data$prostate)

data$TIME.Breast <- data$lenfy_breast
data$TIME.Colorectal <- data$lenfy_crc
data$TIME.Lung <- data$lenfy_lung
data$TIME.Prostate <- data$lenfy_prostate


data$AGE.Breast <- data$age_recruitment + data$lenfy_breast
data$AGE.Breast[!is.na(data$age_breast)] <- data$age_breast[!is.na(data$age_breast)]

data$AGE.Colorectal <- data$age_recruitment + data$lenfy_crc
data$AGE.Colorectal[!is.na(data$age_crc)] <- data$age_crc[!is.na(data$age_crc)]

data$AGE.Lung <- data$age_recruitment + data$lenfy_lung
data$AGE.Lung[!is.na(data$age_lung)] <- data$age_lung[!is.na(data$age_lung)]

data$AGE.Prostate <- data$age_recruitment + data$lenfy_prostate
data$AGE.Prostate[!is.na(data$age_prostate)] <- data$age_prostate[!is.na(data$age_prostate)]

saveRDS(data,"data/data_SCHS_n21694_common_scoresum_22020906.rds")

# ANALYSIS

library(stringr)
library(ggplot2)
library(gridExtra)
library(ggpubr)
library(reshape)
library(scales)
library(VennDiagram)
library(grid)
library(cowplot)
library(lemon)
library(irr)
library(pROC)
library(survival)
library(survAUC)
# setwd("R:/HG/HG7/Private/Datasets/SCHS From Kar Seng/projects/2022-common_cancers")
setwd("/Volumes/research/HG/HG7/Private/Datasets/SCHS From Kar Seng/projects/2022-common_cancers")
data <- readRDS("data/data_SCHS_n21694_common_scoresum_22020906.rds")


form <- function(x){
 format(x,big.mark = ",",big.interval = 3L)
}
form.2 <- function(var,dp=2){
 str_trim(format(round(var,dp),nsmall=dp))
}
form.ci <- function(var,ci,dp=2){
 paste0(form.2(var)," (",form.2(ci[,1])," - ",form.2(ci[,2]),")")
}

form.ci1 <- function(var,ci,dp=2){
 paste0(form.2(var)," (",form.2(ci[1])," - ",form.2(ci[2]),")")
}

form.e<- function(var,dp=2){
 str_trim(formatC(var,digits=dp,format="E"))
}

## STABLE 1

common <- read.csv("data/PGS_proportion_of_variants_missing.csv")
type <- read.csv("data/pgs_all_metadata_score.csv")

common$simplified <- tolower(common$Reported.Trait)
common$simplified[str_detect(common$simplified,"breast")] <- "Breast"
common$simplified[str_detect(common$simplified,"lung")] <- "Lung"
common$simplified[str_detect(common$simplified,"colorectal")] <- "Colorectal"
common$simplified[str_detect(common$simplified,"prostate")] <- "Prostate"

common <- merge(common,type[,c("Polygenic.Score..PGS..ID","PGS.Development.Method","Type.of.Variant.Weight","Original.Genome.Build")],by.x="PGS.ID",by.y="Polygenic.Score..PGS..ID")

common$simplified.type <- common$Type.of.Variant.Weight
common$simplified.type[common$Type.of.Variant.Weight%in%c("beta","beta_Cox","ln(OR)","log(OR)","PHS log(HR)")] <- "Beta/log(OR)"
common$simplified.type[str_detect(tolower(common$Type.of.Variant.Weight),"variance")] <- "inverse-variance weighting"
common$simplified.type[str_detect(tolower(common$Type.of.Variant.Weight),"unweighted")] <- "unweighted"

temp <- common$Ancestry.Distribution.......PGS.Evaluation
common$simplified.ancestry <- "Not East Asian"
common$simplified.ancestry[str_detect(temp,"East Asian")] <- "East Asian"

common$EastAsian.percent <- 0
common$EastAsian.percent[str_detect(temp,"East Asian:100")] <- 100
common$EastAsian.percent[str_detect(temp,"East Asian:33.3")] <- 33.3
common$EastAsian.percent[str_detect(temp,"East Asian:50")] <- 50
common$EastAsian.percent[str_detect(temp,"East Asian:20")] <- 20
common$EastAsian.percent[str_detect(temp,"East Asian:12.5")] <- 12.5


scores.list <- colnames(data)[str_detect(colnames(data),"SCORE")]
temp <- as.character(sapply(scores.list,function(x) str_split(x,"_")[[1]][2]))

common <- common[!str_detect(common$Reported.Trait,"Hyperplasia"),]

scores.list. <- list()

cancer.list <- c("Breast","Prostate","Colorectal","Lung")

for(CANCER in cancer.list){
 scores.list.[[CANCER]] = scores.list[temp%in%common$PGS.ID[common$simplified==CANCER & common$simplified.type%in%c("Beta/log(OR)","NR")]]
}

common <- common[common$simplified.type%in%c("Beta/log(OR)","NR"),]
sex.list = list("Colorectal"=c(2,1), "Breast"=c(2),"Lung"=c(2,1),"Prostate"=c(1))
year.list <- list("5yr"=5,"10yr"=10)

column.select <- c("PGS.ID","S.Name","Reported.Trait",
 "Number.of.Variants","Number.valid.predictors","Percentage.Missing.Predictors","Type.of.Variant.Weight" ,"Original.Genome.Build","Ancestry.Distribution.......PGS.Evaluation",
 "Ancestry.Distribution.......Score.Development.Training",
 "Publication..PMID.","Publication..doi.")

write.csv(common[common$simplified.type%in%c("Beta/log(OR)","NR"),column.select],"output/sTable2_PGS_details_revision.csv",row.names = F)

table(common$simplified)


common[!common$simplified.type%in%c("Beta/log(OR)","NR"),c("Type.of.Variant.Weight","simplified")]

table(common$Ancestry.Distribution.......Score.Development.Training)
table(common$EastAsian.percent)

temp <- common[common$EastAsian.percent!=0,c("PGS.ID","simplified")]
table(temp$simplified)

common[common$Ancestry.Distribution.......Score.Development.Training%in%c("East Asian:100","African:100"),c("PGS.ID","simplified")]

Breast Colorectal Lung Prostate
 85 22 11 37

## Distribution in males and females

### Means and SDs of PGSs

meansd <- function(VAR1,SEX,ETHNICITY="1.Chinese",DATA){
 n <- length(DATA[,VAR1])
 mu <- form.e(mean(DATA[,VAR1]),dp=3)
 sd <- form.e(sd(DATA[,VAR1]),dp=3)
 temp <- as.data.frame(t(c(VAR1,SEX,ETHNICITY,n,mu,sd)))
 colnames(temp) <- c("PRS","Sex","Ethnicity","N_control","Mean_control","SD_control")
 return(temp)
}

temp1 = NULL
for(VAR1 in scores.list){
 temp.a <- meansd(VAR1,SEX="ALL",ETHNICITY="1.Chinese",DATA=data)
 temp.m <- meansd(VAR1,SEX="MALE",ETHNICITY="1.Chinese",DATA=data[data$V9_gender==1,])
 temp.f <- meansd(VAR1,SEX="FEMALE",ETHNICITY="1.Chinese",DATA=data[data$V9_gender==2,])
 temp1 <- rbind(temp1,temp.a,temp.m,temp.f)
}

temp1$PGS.ID <- str_replace(temp1$PRS,"SCORESUM_","")
temp1. <- merge(temp1,common[,c("PGS.ID","Reported.Trait","simplified","Number.of.Variants","Percentage.Missing.Predictors","simplified.type","simplified.ancestry","EastAsian.percent")],by="PGS.ID")

write.csv(temp1.,"data/Common_cancers_mean_sd_revision.csv",row.names = F)

### Distribution for male and females by cases status

dis.table <- function(DATA,COL.PHENO,COL.TIME,NO.YEAR=20,VAR.LIST,MEAN.SD){
 out = NULL
 DATA$PHENO_0 <- DATA[,COL.PHENO]
 DATA$TIME <- DATA[,COL.TIME]
 DATA <- DATA[DATA$PHENO_0%in%c(0,1) & !is.na(DATA$TIME),]
 DATA$PHENO_0[DATA$TIME >= NO.YEAR] <- 0
 DATA$TIME[DATA$TIME >= NO.YEAR] <- NO.YEAR

 for(SCORE in VAR.LIST){
 VAR1 <- DATA[,SCORE]
 mu <- MEAN.SD$Mean_control[MEAN.SD$PRS==SCORE]
 std <- MEAN.SD$SD_control[MEAN.SD$PRS==SCORE]
 VAR1. <- (VAR1-mu)/std
 DATA <- cbind(DATA,VAR1.)
 SD.VAR <- paste0("SD_",as.character(str_split(SCORE,"_")[[1]][2]))
 colnames(DATA)[ncol(DATA)] <- SD.VAR

 noncase = DATA[DATA$PHENO_0==0,SD.VAR]
 case = DATA[DATA$PHENO_0==1,SD.VAR]

 temp <- t.test(case,noncase)
 mean.case = temp$estimate[1]
 mean.noncase = temp$estimate[2]
 sd.case = sd(case)
 sd.noncase = sd(noncase)

 p.value = temp$p.value
 est = temp$estimate[1] - temp$estimate[2]
 ci = temp$conf.int[1:2]

 temp.out <- as.data.frame(t(c(SD.VAR,length(noncase),length(case),
 paste0(form.2(mean.noncase,dp=3)," (",form.2(sd.noncase,dp=3),")"),
 paste0(form.2(mean.case,dp=3)," (",form.2(sd.case,dp=3),")"),
 form.ci1(est,ci,dp=3),form.e(p.value))))

 colnames(temp.out) <- c("PRS","Nnoncases","Ncase",
 "MeanSD_noncase","MeanSD_case",
 " MeanDifference_95%CI","Pvalue_ttest")
 out <- rbind(out,temp.out)
 }
 return(as.data.frame(out))
}

mean.sd <- read.csv("data/Common_cancers_mean_sd_revision.csv")

temp1 = list()
temp1. = NULL
for(CANCER in cancer.list){
 for(SEX in sex.list[[CANCER]]){
 temp <- dis.table(DATA=data[data$V9_gender==SEX,],
 COL.PHENO=paste0("PHENO.",CANCER),
 COL.TIME=paste0("TIME.",CANCER),
 VAR.LIST=scores.list.[[CANCER]],
 MEAN.SD=mean.sd[mean.sd$Sex==c("MALE","FEMALE")[SEX],],
 NO.YEAR=20)
 temp$SEX <- c("MALE","FEMALE")[SEX]
 temp$PHENO <- CANCER
 temp1[[CANCER]][[c("MALE","FEMALE")[SEX]]] <- temp

 temp1. <- rbind(temp1.,temp)

 }
}

OUTPUT.FILE = "Distribution ttest"

temp1.$PGS.ID <- str_replace(temp1.$PRS,"SD_","")
write.csv(temp1.,paste0("output/",OUTPUT.FILE,"_20yr","_revision.csv"),row.names = F)

## Absolute risk

Age-specific (5 year bands) female breast cancer incidence rates in the period 2013-2017, from the Singapore Cancer Registry, and age-specific mortality rates (females only) in 2016, from the Department of Statistics (Singapore) were used in the estimation of absolute risk (National Registry of Diseases Office, 2017; Department of Statistics, 2017). Incidence rates are ethnicity specific while mortality rates are based on all females.

Age- (5 year bands) and gender-specific colon cancer incidence rates in the period 2013-2017, from the Singapore Cancer Registry, and age- and gender-specific mortality rates in 2016, from the Department of Statistics (Singapore) were used in the estimation of absolute risk (National Registry of Diseases Office, 2017; Department of Statistics, 2017). Incidence rates are ethnicity specific while mortality rates are based only by gender.

library(numDeriv)
output.absolute.risk <- function(PRS,MEAN.SD,SEX,ETHNICITY,out.file.name,input.incidence.file.name){
 print(PRS)
 # Generate absolute and lifetime risk for each of this percentile from age 0-80

 incidence.read0 <- read.table(input.incidence.file.name, header = T, sep = ",")

 # SDs for ethnicity
 if(ETHNICITY=="ALL") ethnicity_msd = "ALL"
 if(ETHNICITY=="CHINESE") ethnicity_msd = "1.Chinese"
 if(ETHNICITY=="MALAY") ethnicity_msd = "2.Malay"
 if(ETHNICITY=="INDIAN") ethnicity_msd = "3.Indian"
 sd = as.numeric(as.character(MEAN.SD[MEAN.SD$PRS==PRS &
 MEAN.SD$Ethnicity==ethnicity_msd &
 MEAN.SD$Sex==SEX,"SD_control"]))

 d = 0.6-0.4
 or = NULL

 seqx = seq(0,100,by=.1)
 for (i in 1:1000){
 u = seqx[i]/100
 v = seqx[i+1]/100
 nu=d*(pnorm(qnorm(1-u)+sd)-pnorm(qnorm(1-v)+sd))
 de=(v-u)*(pnorm(qnorm(0.6)+sd)-pnorm(qnorm(0.4)+sd))
 or= c(or,nu/de)
 }

 #The area under the curve (i.e the normal distribution curve, hence the use of pnorm) gives the proportion of the population in any risk group.

 lower = seq(0,99.9,by=.1)
 upper = lower+.1
 name=paste(lower, "-", upper, "%", sep = "")
 prop = (upper - lower)/100

 or = cbind(name, round(or,4),prop)
 colnames(or)=c("PCT", "OR", "Nprob")

 beta.read = or

 # Input for
 if(ETHNICITY == "ALL") incidence.read <- incidence.read0[,c("t","INCIDENCE","DEATH_INCIDENCE")]
 if(ETHNICITY != "ALL"){
 incidence.read <- incidence.read0[,c("t",paste0("INCIDENCE_",ETHNICITY),"DEATH_INCIDENCE")]
 }

 colnames(incidence.read) <- c("t","INCIDENCE","DEATH_INCIDENCE")

 n.prs = dim(beta.read)[1]
 tau = as.numeric(beta.read[,3])
 beta.g = log(as.numeric(beta.read[,2]))
 prs.g = beta.read[,"PCT"]
 incidence = incidence.read[,"INCIDENCE"]/100000
 mortality = incidence.read[,"DEATH_INCIDENCE"]/1000

 Sg = lambda_g = AR_g = AR5_g = matrix(NA, nrow = 85, ncol = n.prs)
 lambda_0 = Sm = rep(NA, length = 85)
 Sg0 = rep(1, length = n.prs)
 beta.g.mat = matrix(rep(beta.g, 85), nrow = 85, ncol = n.prs, byrow = T)
 beta.g.mat[1:20,] = 0

 for (t in 1:85){

 numerator = incidence[t]*sum(tau*Sg0)
 denominator = sum(tau*exp(beta.g.mat[t,])*Sg0)
 lambda_0[t] = numerator/denominator

 lambda_g[t,] = lambda_0[t]*exp(beta.g.mat[t,])
 if (t == 1) {Sg[t,] = exp(-lambda_g[1,])} else {Sg[t,] = exp(-apply(lambda_g[1:t,], 2, "sum"))}

 Sm[t] = exp(-sum(mortality[1:t]))
 Sg0 = Sg[t,]

 if (t==1) {AR_g[t,] = lambda_0[t]*exp(beta.g.mat[t,])*Sg[t,]*Sm[t]}
 else {AR_g[t,] = apply(as.matrix(lambda_0[1:t]*Sm[1:t])%*%exp(beta.g.mat[t,])*Sg[1:t,],2, "sum")}

 if (t >= 30){
 AR5_g[t-5,] = (AR_g[t,]- AR_g[t-5,])/(Sg[t-5,]*Sm[t-5])
 }
 }

 AR_g = as.data.frame(cbind(c(1:85), AR_g))
 colnames(AR_g) <- c("AGE",lower)
 saveRDS(AR_g, paste0(out.file.name,PRS,"_",SEX,"_",ETHNICITY,"_85years.rds"))

 AR5_g = as.data.frame(cbind(c(1:85), AR5_g))
 AR5_g[is.na(AR5_g)]=0
 colnames(AR5_g) <- c("AGE",lower)
 saveRDS(AR5_g, paste0(out.file.name,PRS,"_",SEX,"_",ETHNICITY,"_5yr.rds"))


}

input.incidence = list()

MEAN.SD = mean.sd = read.csv("data/Common_cancers_mean_sd_revision.csv")
ETHNICITY = "CHINESE"

for(CANCER in c("Breast","Colorectal","Lung","Prostate")){

 out.file.name = paste0("data/absolute_risk_tables_",CANCER,"_revision/")
 dir.create(out.file.name)
 for(SEX in sex.list[[CANCER]]){

 input.incidence[[CANCER]][[c("MALE","FEMALE")[SEX]]] = paste0( "data/Incidence_mortality/",CANCER,"_",c("male","female")[SEX],"_2013-2017-5yr.csv")

 for(VAR1 in scores.list.[[CANCER]]){
 output.absolute.risk(PRS=VAR1,
 MEAN.SD=MEAN.SD,
 SEX=c("MALE","FEMALE")[SEX],
 ETHNICITY=ETHNICITY,
 out.file.name=out.file.name,
 input.incidence.file.name=input.incidence[[CANCER]][[c("MALE","FEMALE")[SEX]]])
 }
 }
}

## Plot absolute risk curves

folder: Incidence_mortality Has .csv files of incident and mortality rates prepared for each cancer type.

e.g. Breast_female_2013-2017-5yr.csv

### Breast

CANCER = "Breast"

out.file.name = paste0("data/absolute_risk_tables_",CANCER,"_revision/")
age.col = "age"
# select 1%,every 5%, 99%

select.column <- seq(0,100,5)
select.column[1] <- 1
select.column[length(select.column)] <- 99

eth.list <- c("CHINESE")
ethnicity <- c("Chinese")

different.limit <- paste0("SCORESUM_",c("PGS000015","PGS000501","PGS000502","PGS000509","PGS000510"))
scores.list1 <- scores.list.[[CANCER]]
SEX= "Female"
plot.out <- list()


yaxis.list <- list("5yr" = c(0,0.01,0.02,0.03,0.04,0.05))

ylab.list <- list("5yr" = "Five-year absolute risk (%)")

for(YEAR in c("5yr")){
 for(SCORE in scores.list1){
 absolute.risk.table <-
 readRDS(paste0(out.file.name,SCORE,"_",toupper(SEX),"_",ETHNICITY,"_",YEAR,".rds"))
 AR5_g <- absolute.risk.table[,c(1,which(colnames(absolute.risk.table)%in%select.column))]
 AR5_g <- as.data.frame(AR5_g[1:75,])
 melted.arg.5 <- melt(AR5_g, id = "AGE")
 colour <- hue_pal()(length(select.column))

 melted.arg.5$value <- as.numeric(melted.arg.5$value)
 p <- ggplot(melted.arg.5, aes(x = AGE, y= value, color = variable))+
 geom_line(data=melted.arg.5, aes(x = AGE, y= value, color = variable)) +
 scale_color_manual(breaks=levels(factor(melted.arg.5$variable)),
 values = colour,
 # labels=c(label),
 guide=guide_legend(override.aes = list(
 color=colour)),
 name=bquote("SCORE \npercentiles")) +
 ylab(label=ylab.list[[YEAR]]) +
 xlab("Age, years") +
 scale_x_continuous(labels = c(seq(20, 70,10)),breaks= c(seq(20, 70,10)), expand = c(0, 0)) +
 guides(color=guide_legend(nrow=4,direction="horizontal")) +
 theme(legend.position="bottom",panel.grid.minor = element_blank())

 if(!SCORE %in% different.limit){
 plot.out[[SCORE]][[SEX]] <- p +
 coord_cartesian(xlim=c(23,77),ylim = c(0,yaxis.list[[YEAR]][length(yaxis.list[[YEAR]])])) +
 scale_y_continuous(labels =format(round(yaxis.list[[YEAR]] *100,1),nsmall=1), breaks = yaxis.list[[YEAR]])
 }
 if(SCORE %in% different.limit){
 plot.out[[SCORE]][[SEX]] <- p +
 coord_cartesian(xlim=c(23,77),ylim = c(0,.25)) +
 scale_y_continuous(labels =format(round(seq(0,0.4,.01)*100,1),nsmall=1), breaks = seq(0,.4,.01))
 }
 }

 output.file = paste0("plot/Absolute_risk_",CANCER,"_",YEAR,"_revision")
 dir.create(output.file)
 for(SCORE in scores.list1){
 png(paste0(output.file,"/",SCORE,".png"),res=300,width=1300,height=1500)
 print(ggarrange(plot.out[[SCORE]][[SEX]],nrow=1,ncol=1,common.legend = T,legend = "bottom"))
 dev.off()
 }

}

### Colorectal

CANCER = "Colorectal"

out.file.name = paste0("data/absolute_risk_tables_",CANCER,"_revision/")
age.col = "age"
# select 1%,every 5%, 99%

select.column <- seq(0,100,5)
select.column[1] <- 1
select.column[length(select.column)] <- 99

eth.list <- c("CHINESE")
ethnicity <- c("Chinese")
label.list <- list()
for(SEX in sex.list[[CANCER]]){
 label.list[[c("Male","Female")[SEX]]] <- paste0("(",LETTERS[SEX],") ",c("Male","Female")[SEX])
}
common[common$PGS.ID%in%c("PGS000150","PGS000375"),]
scores.list1 <- scores.list.[[CANCER]][!scores.list.[[CANCER]]%in%paste0("SCORESUM_",c("PGS000150"))]

yaxis.list <- list("5yr" = c(0,0.01,0.02,0.03))
for(YEAR in c("5yr")){

 plot.out <- list()
 for(SCORE in scores.list1){
 # SCORE = "SCORE_PGS000785"
 for(SEX in c("Male","Female")){

 absolute.risk.table <-
 readRDS(paste0(out.file.name,SCORE,"_",toupper(SEX),"_",ETHNICITY,"_",YEAR,".rds"))
 AR5_g <- absolute.risk.table[,c(1,which(colnames(absolute.risk.table)%in%select.column))]
 AR5_g <- as.data.frame(AR5_g[1:75,])
 melted.arg.5 <- melt(AR5_g, id = "AGE")
 melted.arg.5$value <- as.numeric(melted.arg.5$value)

 colour <- hue_pal()(length(select.column))

 plot.out[[SCORE]][[SEX]] <-
 ggplot(melted.arg.5, aes(x = AGE, y= value, color = variable))+
 geom_line(data=melted.arg.5, aes(x = AGE, y= value, color = variable)) +
 scale_color_manual(breaks=levels(factor(melted.arg.5$variable)),
 values = colour,
 # labels=c(label),
 guide=guide_legend(override.aes = list(
 color=colour)),
 name=bquote("SCORE percentiles")) +
 coord_cartesian(xlim=c(23,77),ylim = c(0,yaxis.list[[YEAR]][length(yaxis.list[[YEAR]])])) +
 ylab(label="Five-year absolute risk (%)") +
 xlab("Age, years") +
 scale_y_continuous(labels =format(round(yaxis.list[[YEAR]]*100,1),nsmall=1), breaks = yaxis.list[[YEAR]])+
 scale_x_continuous(labels = c(seq(20, 70,10)),breaks= c(seq(20, 70,10)), expand = c(0, 0)) +
 ggtitle(label.list[[SEX]]) +
 guides(color=guide_legend(nrow=2,direction="horizontal")) +
 theme(legend.position="bottom",panel.grid.minor = element_blank())

 }
 }
 output.file = paste0("plot/Absolute_risk_",CANCER,"_",YEAR,"_revision")
 dir.create(output.file)
 for(SCORE in scores.list1){
 png(paste0(output.file,"/",SCORE,".png"),res=300,width=2500,height=1500)
 print(ggarrange(plot.out[[SCORE]][["Male"]],plot.out[[SCORE]][["Female"]],nrow=1,ncol=2,common.legend = T,legend = "bottom"))
 dev.off()
 }
}

### Lung

CANCER = "Lung"

out.file.name = paste0("data/absolute_risk_tables_",CANCER,"_revision/")
age.col = "age"
# select 1%,every 5%, 99%

select.column <- seq(0,100,5)
select.column[1] <- 1
select.column[length(select.column)] <- 99

eth.list <- c("CHINESE")
ethnicity <- c("Chinese")
label.list <- list()
for(SEX in sex.list[[CANCER]]){
 label.list[[c("Male","Female")[SEX]]] <- paste0("(",LETTERS[SEX],") ",c("Male","Female")[SEX])
}
scores.list1 <- scores.list.[[CANCER]]

yaxis.list <- list("5yr" = seq(0,0.07,.01))
for(YEAR in c("5yr")){

 plot.out <- list()
 for(SCORE in scores.list1){
 # SCORE = "SCORE_PGS000785"
 for(SEX in c("Male","Female")){

 absolute.risk.table <-
 readRDS(paste0(out.file.name,SCORE,"_",toupper(SEX),"_",ETHNICITY,"_",YEAR,".rds"))
 AR5_g <- absolute.risk.table[,c(1,which(colnames(absolute.risk.table)%in%select.column))]
 AR5_g <- as.data.frame(AR5_g[1:75,])
 melted.arg.5 <- melt(AR5_g, id = "AGE")
 melted.arg.5$value <- as.numeric(melted.arg.5$value)

 colour <- hue_pal()(length(select.column))

 plot.out[[SCORE]][[SEX]] <-
 ggplot(melted.arg.5, aes(x = AGE, y= value, color = variable))+
 geom_line(data=melted.arg.5, aes(x = AGE, y= value, color = variable)) +
 scale_color_manual(breaks=levels(factor(melted.arg.5$variable)),
 values = colour,
 # labels=c(label),
 guide=guide_legend(override.aes = list(
 color=colour)),
 name=bquote("SCORE percentiles")) +
 coord_cartesian(xlim=c(23,77),ylim = c(0,yaxis.list[[YEAR]][length(yaxis.list[[YEAR]])])) +
 ylab(label="Five-year absolute risk (%)") +
 xlab("Age, years") +
 scale_y_continuous(labels =format(round(yaxis.list[[YEAR]]*100,1),nsmall=1), breaks = yaxis.list[[YEAR]])+
 scale_x_continuous(labels = c(seq(20, 70,10)),breaks= c(seq(20, 70,10)), expand = c(0, 0)) +
 ggtitle(label.list[[SEX]]) +
 guides(color=guide_legend(nrow=2,direction="horizontal")) +
 theme(legend.position="bottom",panel.grid.minor = element_blank())

 }
 }
 output.file = paste0("plot/Absolute_risk_",CANCER,"_",YEAR,"_revision")
 dir.create(output.file)
 for(SCORE in scores.list1){
 png(paste0(output.file,"/",SCORE,".png"),res=300,width=2500,height=1500)
 print(ggarrange(plot.out[[SCORE]][["Male"]],plot.out[[SCORE]][["Female"]],nrow=1,ncol=2,common.legend = T,legend = "bottom"))
 dev.off()
 }
}

### Prostate

CANCER = "Prostate"

out.file.name = paste0("data/absolute_risk_tables_",CANCER,"_revision/")
age.col = "age"
# select 1%,every 5%, 99%

select.column <- seq(0,100,5)
select.column[1] <- 1
select.column[length(select.column)] <- 99

eth.list <- c("CHINESE")
ethnicity <- c("Chinese")

different.limit <- paste0("SCORESUM_",c("PGS000577","PGS000578","PGS000579","PGS000580","PGS000581",
 "PGS000582","PGS000585","PGS000586","PGS000589","PGS000591","PGS001805"))
scores.list1 <- scores.list.[[CANCER]]
SEX= "Male"
plot.out <- list()


yaxis.list <- list("5yr" = seq(0,0.05,.01))

ylab.list <- list("5yr" = "Five-year absolute risk (%)")

for(YEAR in c("5yr")){
 for(SCORE in scores.list1){
 absolute.risk.table <-
 readRDS(paste0(out.file.name,SCORE,"_",toupper(SEX),"_",ETHNICITY,"_",YEAR,".rds"))
 AR5_g <- absolute.risk.table[,c(1,which(colnames(absolute.risk.table)%in%select.column))]
 AR5_g <- as.data.frame(AR5_g[1:75,])
 melted.arg.5 <- melt(AR5_g, id = "AGE")
 colour <- hue_pal()(length(select.column))

 melted.arg.5$value <- as.numeric(melted.arg.5$value)
 p <- ggplot(melted.arg.5, aes(x = AGE, y= value, color = variable))+
 geom_line(data=melted.arg.5, aes(x = AGE, y= value, color = variable)) +
 scale_color_manual(breaks=levels(factor(melted.arg.5$variable)),
 values = colour,
 # labels=c(label),
 guide=guide_legend(override.aes = list(
 color=colour)),
 name=bquote("SCORE \npercentiles")) +
 ylab(label=ylab.list[[YEAR]]) +
 xlab("Age, years") +
 scale_x_continuous(labels = c(seq(20, 70,10)),breaks= c(seq(20, 70,10)), expand = c(0, 0)) +
 guides(color=guide_legend(nrow=4,direction="horizontal")) +
 theme(legend.position="bottom",panel.grid.minor = element_blank())

 if(!SCORE %in% different.limit){
 plot.out[[SCORE]][[SEX]] <- p +
 coord_cartesian(xlim=c(23,77),ylim = c(0,yaxis.list[[YEAR]][length(yaxis.list[[YEAR]])])) +
 scale_y_continuous(labels =format(round(yaxis.list[[YEAR]] *100,1),nsmall=1), breaks = yaxis.list[[YEAR]])
 }
 if(SCORE %in% different.limit){
 plot.out[[SCORE]][[SEX]] <- p +
 coord_cartesian(xlim=c(23,77),ylim = c(0,.16)) +
 scale_y_continuous(labels =format(round(seq(0,0.16,.01)*100,1),nsmall=1), breaks = seq(0,.16,.01))
 }
 }

 output.file = paste0("plot/Absolute_risk_",CANCER,"_",YEAR,"_revision")
 dir.create(output.file)
 for(SCORE in scores.list1){
 png(paste0(output.file,"/",SCORE,".png"),res=300,width=1300,height=1500)
 print(ggarrange(plot.out[[SCORE]][[SEX]],nrow=1,ncol=1,common.legend = T,legend = "bottom"))
 dev.off()
 }

}

## Adding absolute risk

stand.all <- function(ETHNICITY,SEX.COL,PRS,DATA,MEAN.SD){
 temp.sd <- rep(NA,nrow(DATA))
 for(i in c(1,2)){
 mu = as.numeric(as.character(MEAN.SD$Mean_control[MEAN.SD$Ethnicity==ETHNICITY &
 MEAN.SD$Sex==c("MALE","FEMALE")[i] &
 MEAN.SD$PRS==PRS]))
 sd = as.numeric(as.character(MEAN.SD$SD_control[MEAN.SD$Ethnicity==ETHNICITY &
 MEAN.SD$Sex==c("MALE","FEMALE")[i] &
 MEAN.SD$PRS==PRS]))

 temp = DATA[DATA[,SEX.COL]==i,PRS]
 temp.sd[DATA[,SEX.COL]==i] <- (temp-mu)/sd
 }
 print(summary(temp.sd))
 return(temp.sd)
}

assign.risk <- function(PERCENTILE,AGE,absolute.risk.table){
 AGE[!is.na(AGE) & AGE>80] <- NA
 PERCENTILE[PERCENTILE==100] <- 99.9

 temp = numeric(length(PERCENTILE))
 for(ind in 1:length(PERCENTILE)){
 if(is.na(AGE[ind])) temp[ind] <- NA
 if(!is.na(AGE[ind])) temp[ind] <- absolute.risk.table[absolute.risk.table[,"AGE"]==AGE[ind],colnames(absolute.risk.table)==PERCENTILE[ind]]
 }

 out <- temp*100
 return(out)
}

### 5-year

ETHNICITY = "CHINESE"
SEX.COL= "V9_gender"
AGE.COL= "age_recruitment"
mean.sd <- read.csv("data/Common_cancers_mean_sd_revision.csv")

dir.create("data/PGS_standardized_revision")

for(CANCER in cancer.list){
 temp.sd <- matrix(NA,nc=length(scores.list.[[CANCER]]),nr=nrow(data))
 colnames(temp.sd) <- paste0(scores.list.[[CANCER]],".SD")
 temp.percentile <- matrix(NA,nc=length(scores.list.[[CANCER]]),nr=nrow(data))
 colnames(temp.percentile) <- paste0(scores.list.[[CANCER]],".percentile")
 temp.absolute.risk <- matrix(NA,nc=length(scores.list.[[CANCER]]),nr=nrow(data))
 colnames(temp.absolute.risk) <- paste0(scores.list.[[CANCER]],".absolute.risk")

 out.file.name = paste0("data/absolute_risk_tables_",CANCER,"_revision/")

 for(SCORE in scores.list.[[CANCER]]){

 if(ETHNICITY=="CHINESE") ethnic = "1.Chinese"
 stand.deviation <- stand.all(ETHNICITY = ethnic, SEX.COL= SEX.COL,PRS =SCORE, DATA = data, MEAN.SD = mean.sd)
 percentile = round(pnorm(stand.deviation) * 100,1)
 age = round(as.numeric(as.character(data[,AGE.COL])))

 absolute.risk <- rep(NA,nrow(data))
 for(i in sex.list[[CANCER]]){
 absolute.risk.table <-
 readRDS(paste0(out.file.name,SCORE,"_",c("MALE","FEMALE")[i],"_",ETHNICITY,"_5yr.rds"))
 absolute.risk[data[,SEX.COL]==i] <- assign.risk(PERCENTILE=percentile[data[,SEX.COL]==i],
 AGE=age[data[,SEX.COL]==i],
 absolute.risk.table=absolute.risk.table)
 }
 temp.sd[,str_detect(colnames(temp.sd),SCORE)] <- stand.deviation
 temp.percentile[,str_detect(colnames(temp.percentile),SCORE)] <- percentile
 temp.absolute.risk[,str_detect(colnames(temp.absolute.risk),SCORE)] <- absolute.risk
 }
 data.out <- cbind(data[,1],temp.sd,temp.percentile,temp.absolute.risk)
 colnames(data.out)[1] <- "FID"
 write.csv(data.out,paste0("data/PGS_standardized_revision/",CANCER,"_standardized_5yr.csv"),row.names = F)
}

## Calibration

### Calculation by decile

library(pROC)
library(glmtoolbox)
for(YEAR in c("5yr")){
 for(CANCER in cancer.list){
 for(SEX in sex.list[[CANCER]]){
 COL.TIME = paste0("TIME.",CANCER)
 COL.STATUS = paste0("PHENO.",CANCER)

 temp <- read.csv(paste0("data/PGS_standardized_revision/",CANCER,"_standardized_",YEAR,".csv"))
 data.abs0 <- merge(data,temp,by="FID")
 data.abs0 <- data.abs0[data.abs0$V9_gender==SEX,]
 data.abs <- data.abs0[data.abs0$age_recruitment>30 & data.abs0$age_recruitment<70 & data.abs0[,COL.STATUS]%in%c(0,1),]
 table(data.abs[,COL.STATUS],useNA = "ifany")

 data.abs$status <- rep(0,nrow(data.abs))
 data.abs$status[data.abs[,COL.STATUS]==1 & !is.na(data.abs[,COL.TIME]) & data.abs[,COL.TIME]<=year.list[[YEAR]]] <- 1

 cal.data = list()

 for(SCORE in scores.list.[[CANCER]]){

 # ALL
 absrisk <- data.abs[,paste0(SCORE,".absolute.risk")]
 print(summary(absrisk))
 expected <- sum(absrisk/100)
 observed <- sum(data.abs$status)
 calibration <- expected/observed
 tmp <- qnorm(.975)*sqrt(1/observed)
 upper <- calibration * exp(tmp)
 lower <- calibration * exp(-1 * tmp)
 calibration. <- paste0(form.2(calibration,dp=2)," (",form.2(lower,dp=2),"\226",form.2(upper,dp=2), ")")

 ## Hosmer-Lemeshow goodness of fit test for logistic regression
 hlmodel <- glm(data.abs$status~absrisk,family="binomial")
 hl.p <- hltest(hlmodel,group=10)$p.value

 ## ROC
 model <- roc(data.abs$status~absrisk)
 temp <- ci.auc(model)
 auc. <- paste0(form.2(temp[2],3)," (",form.2(temp[1],3)," \226 ",form.2(temp[3],3),")")

 temp.all <- as.data.frame(t(c(SCORE,"ALL",0,
 expected,observed,calibration.,auc.,
 calibration,lower,upper,
 temp[2],temp[1],temp[3],
 length(absrisk),
 hl.p)))
 colnames(temp.all) <- c("PGS","Decile","Order",
 "Expected","Observed","Calibration","AUC",
 "cal","cal.lower","cal.upper",
 "auc","auc.lower","auc.upper",
 "N","HL.p")
 # By decile
 print(summary(as.numeric(data.abs[,paste0(SCORE,".absolute.risk")])))
 threshold <- quantile(data.abs[,paste0(SCORE,".absolute.risk")], seq(0,1,.1))
 decile <- cut(data.abs[,paste0(SCORE,".absolute.risk")],threshold,include.lowest=T,ordered_result=T)
 decile.list <- levels(decile)

 temp.out. = NULL
 for(DEC in 1:length(decile.list)){
 tmp.d <- data.abs[decile==decile.list[DEC],]
 absrisk <- tmp.d[,paste0(SCORE,".absolute.risk")]
 print(summary(absrisk))
 expected <- sum(absrisk/100)

 observed <- sum(tmp.d$status)
 calibration <- expected/observed
 tmp <- qnorm(.975)*sqrt(1/observed)
 upper <- calibration * exp(tmp)
 lower <- calibration * exp(-1 * tmp)
 calibration. <- paste0(form.2(calibration,dp=2)," (",form.2(lower,dp=2),"\226",form.2(upper,dp=2), ")")


 if(observed>0){
 model <- roc(tmp.d$status~absrisk)
 temp <- ci.auc(model)*100
 auc. <- paste0(form.2(temp[2],3)," (",form.2(temp[1],3)," \226 ",form.2(temp[3],3),")")
 temp.out <- c(SCORE,as.character(decile.list[DEC]),DEC,
 expected,observed,calibration.,auc.,
 calibration,lower,upper,
 temp[2],temp[1],temp[3],
 length(absrisk))
 }
 if(observed==0){
 temp.out <- c(SCORE,as.character(decile.list[DEC]),DEC,
 expected,observed,rep(NA,8),
 length(absrisk))
 }
 temp.out. <- rbind(temp.out.,temp.out)

 }
 temp.out. <- cbind(temp.out.,"")
 colnames(temp.out.) <- c("PGS","Decile","Order",
 "Expected","Observed","Calibration","AUC",
 "cal","cal.lower","cal.upper",
 "auc","auc.lower","auc.upper",
 "N","HL.p")
 cal.data[[SCORE]] <- rbind(temp.all,temp.out.)
 }

 cal.data. <- do.call(rbind, cal.data)
 # print(cal.data.)
 write.csv(cal.data.,paste0("output/Calibration_",CANCER,"_",c("MALE","FEMALE")[SEX],"_",YEAR,"_revision.csv"),row.names = F)
 }
 }
}

### Slope and intercept - linear model

slope.int = list()
for(YEAR in c("5yr")){
 for(CANCER in cancer.list){
 for(SEX in sex.list[[CANCER]]){
 cal.data. <- read.csv(paste0("output/Calibration_",CANCER,"_",c("MALE","FEMALE")[SEX],"_",YEAR,"_revision.csv"))

 cal.data.$x <- cal.data.$Expected/cal.data.$N
 cal.data.$y <- cal.data.$Observed/cal.data.$N

 temp.data <- cal.data.[cal.data.$Order!=0,]
 temp.out=NULL
 for(SCORE in scores.list.[[CANCER]]){
 temp <- temp.data[temp.data$PGS==SCORE,]
 if(length(which(temp.data$PGS==SCORE))==0) print(SCORE)
 fit <- lm(y~x,data=temp)
 coef <- as.data.frame(summary(fit)$coef[,1:2])
 coef$lower <- coef$Estimate - qnorm(.975) * coef$Estimate
 coef$upper <- coef$Estimate + qnorm(.975) * coef$Estimate
 temp1 <- cbind(SCORE,paste0(c("Intercept=","Slope="),
 form.2(coef$Estimate,dp=2),
 " (",form.2(coef$lower,dp=2),", ",
 form.2(coef$upper,dp=2),")"))
 temp.out <- rbind(temp.out,temp1)
 }
 colnames(temp.out) <- c("PGS","Text")
 slope.int[[CANCER]][[YEAR]] <- temp.out
 }
 }
}

### Plot by decile

Estimates and 95% CI of the calibration slope and intercept are reported based on a linear regression of the decile-specific observed proportion of cases within 5 years and the average of the predicted 5-year absolute risk.

YEAR="5yr"
CANCER = "Breast"

axis.list = list()
axis.list[["Breast"]][["5yr"]] = c(0,4)
axis.list[["Colorectal"]][["5yr"]] = c(0,2)
axis.list[["Lung"]][["5yr"]] = c(0,2)
axis.list[["Prostate"]][["5yr"]] = c(0,4)

for(YEAR in c("5yr")){
 for(CANCER in cancer.list){
 for(SEX in sex.list[[CANCER]]){
 cal.data. <- read.csv(paste0("output/Calibration_",CANCER,"_",c("MALE","FEMALE")[SEX],"_",YEAR,"_revision.csv"))

 dir.create(paste0("plot/Calibration_plot_",CANCER,"_",c("MALE","FEMALE")[SEX],"_",YEAR,"_revision"))

 dataplot <- as.data.frame(cal.data.[cal.data.$Order!=0,c("PGS","Decile","Order","Observed","Expected","N")])
 dataplot$Order <- factor(dataplot$Order,1:10)
 for(i in 4:6){
 dataplot[,i] <- as.numeric(as.character(dataplot[,i]))
 }

 dataplot$proportion <- dataplot$Observed/dataplot$N

 dataplot$upper = dataplot$lower = rep(NA,nrow(dataplot))
 for(i in 1:nrow(dataplot)){
 dataplot[i,c("lower","upper")] <- as.numeric(prop.test(dataplot$Observed[i],dataplot$N[i],correct=FALSE)$conf.int[1:2])
 }

 dataplot$proportion.o <-dataplot$proportion * 100
 dataplot$upper <- dataplot$upper *100
 dataplot$lower <- dataplot$lower *100
 dataplot$proportion.e <- dataplot$Expected/dataplot$N *100

 for(SCORE in scores.list.[[CANCER]]){
 axis <- axis.list[[CANCER]][[YEAR]]
 slope.inter <- as.data.frame(slope.int[[CANCER]][[YEAR]])
 slope.inter <- slope.inter[slope.inter$PGS==SCORE,]
 slope.inter$y <- c(0.1,.4)
 if(CANCER %in% c("Colorectal","Lung")) slope.inter$y <- c(0.1,.2)
 slope.inter$x <- c(axis[2],axis[2])

 title <- as.character(sapply(SCORE,function(x){str_split(x,"_")[[1]][2]}))
 p <- ggplot(data=dataplot[dataplot$PGS==SCORE,],
 aes(x=as.numeric(proportion.e),y=as.numeric(proportion.o),color=factor(Order))) +
 geom_abline(intercept = 0, slope = 1,color="Grey",linetype="dashed") +
 geom_point() +
 geom_segment(aes(x=as.numeric(proportion.e),xend=as.numeric(proportion.e),y=lower,yend=upper)) +
 geom_text(data=slope.inter,aes(x=x,y=y,label=Text),color="black",hjust=1) +

 coord_cartesian(xlim = axis,ylim=axis) +
 scale_color_manual(breaks=1:10,values=hue_pal()(10),labels=dataplot$Decile[dataplot$PGS==SCORE],
 guide=guide_legend(nrow = 3)) +
 labs(x="Expected (%)",y="Observed (%)",title = title,color="Deciles") +
 theme_light() +
 theme(legend.position = "bottom",legend.text = element_text(size = 8))

 png(paste0("plot/Calibration_plot_",CANCER,"_",c("MALE","FEMALE")[SEX],"_",YEAR,"_revision/",SCORE,".png"),res=120,width=600,height=650)
 print(p)
 dev.off()
 }
 }
 }

}

Furthermore, we sought to estimate the percentage of individuals in the general population who can be predicted to have an at least twofold elevated risk of cancer, a risk level comparable to the risk associated with many moderate-penetrance mutations in known cancer predisposition genes that are currently included in clinical genetic testing.

## Survival, AUC and OR/HR

### Per SD, High risk individual

auc.table <- function(DATA,COL.PHENO,COL.TIME,NO.YEAR=5,VAR.LIST,MEAN.SD){
 out = NULL
 DATA$PHENO_0 <- DATA[,COL.PHENO]
 DATA$TIME <- DATA[,COL.TIME]
 DATA <- DATA[DATA$PHENO_0%in%c(0,1) & !is.na(DATA$TIME),]
 DATA$PHENO_0[DATA$TIME >= NO.YEAR] <- 0
 DATA$TIME[DATA$TIME >= NO.YEAR] <- NO.YEAR

 for(SCORE in VAR.LIST){
 VAR1 <- DATA[,SCORE]
 mu <- MEAN.SD$Mean_control[MEAN.SD$PRS==SCORE]
 std <- MEAN.SD$SD_control[MEAN.SD$PRS==SCORE]
 VAR1. <- (VAR1-mu)/std
 DATA <- cbind(DATA,VAR1.)
 SD.VAR <- paste0("SD_",as.character(str_split(SCORE,"_")[[1]][2]))
 colnames(DATA)[ncol(DATA)] <- SD.VAR

 # GLM for CI unadjusted
 fit <- glm(formula(paste0("PHENO_0~",SD.VAR)),data=DATA,family="binomial")
 unadj <- roc(DATA$PHENO_0~predict(fit,DATA,type="response"))
 unadj2 <- ci(unadj)

 # Cox per SD unadjusted
 cox <- coxph(formula(paste0("Surv(TIME,PHENO_0)~",SD.VAR)),data=DATA)
 unadj.p.value <- summary(cox)$coefficients[1,5]
 coeff <- summary(cox)$conf.int
 unadj.HR.cox <- coeff[1,1]
 unadj.CI <- c(coeff[1,3],coeff[1,4])
 unadj.ph.pvalue <- cox.zph(cox)$table[1,3]


 # GLM for CI
 fit <- glm(formula(paste0("PHENO_0~",SD.VAR,"+age_recruitment")),data=DATA,family="binomial")
 temp <- roc(DATA$PHENO_0~predict(fit,DATA,type="response"))
 temp2 <- ci(temp)

 # Cox per SD
 cox <- coxph(formula(paste0("Surv(TIME,PHENO_0)~",SD.VAR,"+age_recruitment")),data=DATA)
 p.value <- summary(cox)$coefficients[1,5]
 coeff <- summary(cox)$conf.int
 HR.cox <- coeff[1,1]
 CI <- c(coeff[1,3],coeff[1,4])
 ph.pvalue <- cox.zph(cox)$table[1,3]


 # output


 temp.out <- as.data.frame(t(c(SD.VAR,
 form.ci1(unadj2[2],unadj2[c(1,3)],dp=3),

 form.ci1(unadj.HR.cox,CI,dp=3),
 form.e(unadj.p.value),form.2(unadj.ph.pvalue,dp=3),

 form.ci1(temp2[2],temp2[c(1,3)],dp=3),
 form.ci1(HR.cox,CI,dp=3),
 form.e(p.value),form.2(ph.pvalue,dp=3),
 form.2(mean(VAR1),dp=3),form.e(sd(VAR1),dp=3),
 nrow(DATA),
 temp2[c(2,1,3)],
 HR.cox,CI)))

 colnames(temp.out) <- c("PRS",
 "unadjusted AUC (95%CI)","unadjusted HR (95%CI)",
 "unadjusted P (Cox HR)","unadjusted P (PH assumption)",
 "AUC (95%CI)",
 "HR (95%CI)","P (Cox HR)","P (PH assumption)",
 "Mean","SD","N",
 "auc","auc.lower","auc.upper",
 "hr","hr.lower","hr.upper")
 out <- rbind(out,temp.out)
 }
 return(as.data.frame(out))
}

mean.sd <- read.csv("data/Common_cancers_mean_sd_revision.csv")


temp1 = list()
temp1. = NULL
for(CANCER in cancer.list){
 for(SEX in sex.list[[CANCER]]){
 temp <- auc.table(DATA=data[data$V9_gender==SEX,],
 COL.PHENO=paste0("PHENO.",CANCER),
 COL.TIME=paste0("TIME.",CANCER),
 VAR.LIST=scores.list.[[CANCER]],
 MEAN.SD=mean.sd[mean.sd$Sex==c("MALE","FEMALE")[SEX],],
 NO.YEAR=20)
 temp$SEX <- c("MALE","FEMALE")[SEX]
 temp$PHENO <- CANCER
 temp1[[CANCER]][[c("MALE","FEMALE")[SEX]]] <- temp

 temp1. <- rbind(temp1.,temp)

 }
}

OUTPUT.FILE = "AUC_common_cancer_scoresum"

temp1.$PGS.ID <- str_replace(temp1.$PRS,"SD_","")
temp1. <- merge(temp1.,common,by="PGS.ID")
write.csv(temp1.,paste0("output/",OUTPUT.FILE,"_SD_labeled_","20yr","_revision.csv"),row.names = F)

### Quintile (3rd ref)

auc.quintile.table <- function(DATA,COL.PHENO,COL.TIME,NO.YEAR=5,VAR.LIST,MEAN.SD){
 out = NULL
 DATA$PHENO_0 <- DATA[,COL.PHENO]
 DATA$TIME <- DATA[,COL.TIME]
 DATA <- DATA[DATA$PHENO_0%in%c(0,1) & !is.na(DATA$TIME),]
 DATA$PHENO_0[DATA$TIME >= NO.YEAR] <- 0
 DATA$TIME[DATA$TIME >= NO.YEAR] <- NO.YEAR
 quintile = NULL
 for(SCORE in VAR.LIST){
 VAR1 <- DATA[,SCORE]
 mu <- MEAN.SD$Mean_control[MEAN.SD$PRS==SCORE]
 std <- MEAN.SD$SD_control[MEAN.SD$PRS==SCORE]
 VAR1. <- (VAR1-mu)/std
 DATA <- cbind(DATA,VAR1.)
 SD.VAR <- paste0("SD_",as.character(str_split(SCORE,"_")[[1]][2]))
 colnames(DATA)[ncol(DATA)] <- SD.VAR

 tmp <- rep(0,nrow(DATA))
 tmp[VAR1.>qnorm(.8)] <- "Q5"
 tmp[VAR1.>qnorm(.6) & VAR1.<=qnorm(.8)] <- "Q4"
 tmp[VAR1.>qnorm(.4) & VAR1.<=qnorm(.6)] <- "Q3"
 tmp[VAR1.>qnorm(.2) & VAR1.<=qnorm(.4)] <- "Q2"
 tmp[VAR1.<=qnorm(.2)] <- "Q1"

 tmp <- factor(tmp,paste0("Q",c(3,1,2,4,5)))

 tmp.con <- as.numeric(as.character(str_replace(tmp,"Q","")))

 DATA <- cbind(DATA,tmp,tmp.con) ## compare to 40-60
 PER.VAR <- paste0("PER_",as.character(str_split(SCORE,"_")[[1]][2]))
 CON.VAR <- paste0("CON_",as.character(str_split(SCORE,"_")[[1]][2]))
 colnames(DATA)[ncol(DATA)-1] <- PER.VAR
 colnames(DATA)[ncol(DATA)] <- CON.VAR
 print(table(DATA[DATA[,"PHENO_0"]==1,PER.VAR]))
 print(table(DATA[DATA[,"PHENO_0"]==1,CON.VAR]))

 cases <- table(DATA[DATA[,"PHENO_0"]==1,PER.VAR])


 # GLM for CI
 fit <- glm(formula(paste0("PHENO_0~",PER.VAR,"+age_recruitment")),data=DATA,family="binomial")
 temp <- roc(DATA$PHENO_0~predict(fit,DATA,type="response"))
 temp2 <- ci(temp)

 # Cox quintile
 cox <- coxph(formula(paste0("Surv(TIME,PHENO_0)~",PER.VAR,"+age_recruitment")),data=DATA)
 p.value <- summary(cox)$coefficients[,5]
 coeff <- summary(cox)$conf.int
 HR.cox <- coeff[,1]
 CI <- cbind(coeff[,3],coeff[,4])
 ph.pvalue <- cox.zph(cox)$table[1,3]

 ci. = character(nrow(CI))
 for(r in 1:nrow(CI)){
 ci.[r] <- form.ci1(HR.cox[r],CI[r,],dp=3)
 }
 tmp1 = NULL
 for(q in 1:length(ci.)){
 tmp1 <- c(tmp1,ci.[q],form.e(p.value[q]))
 }
 tmp1.names <- paste0(rep(c("HR (95%CI)","P (Cox HR)"),4),"_Q",kronecker(c(1,2,4,5),rep(1,2)))

 # p-trend
 cox.con <- coxph(formula(paste0("Surv(TIME,PHENO_0)~",CON.VAR,"+age_recruitment")),data=DATA)
 p.trend <- summary(cox.con)$coefficients[,5]

 temp.out <- as.data.frame(t(c(SD.VAR,form.ci1(temp2[2],temp2[c(1,3)],dp=3),
 tmp1,form.e(p.trend)[1],form.2(ph.pvalue,dp=3),
 form.2(mean(VAR1),dp=3),form.e(sd(VAR1),dp=3),
 nrow(DATA),
 temp2[c(2,1,3)],
 cases
 )))

 # output
 colnames(temp.out) <- c("PRS","AUC (95%CI)",
 tmp1.names,"age at recruitment","P (Cox HR)_age","Ptrend","P (PH assumption)",
 "Mean","SD","N",
 "auc","auc.lower","auc.upper",
 names(cases)
 )

 out <- rbind(out,temp.out)
 }
 return(as.data.frame(out))
}

mean.sd <- read.csv("data/Common_cancers_mean_sd_revision.csv")

temp1 = list()
temp1. = NULL
for(CANCER in cancer.list){
 for(SEX in sex.list[[CANCER]]){
 temp <- auc.quintile.table(DATA=data[data$V9_gender==SEX,],
 COL.PHENO=paste0("PHENO.",CANCER),
 COL.TIME=paste0("TIME.",CANCER),
 VAR.LIST=scores.list.[[CANCER]],
 MEAN.SD=mean.sd[mean.sd$Sex==c("MALE","FEMALE")[SEX],],
 NO.YEAR=20)


 temp$SEX <- c("MALE","FEMALE")[SEX]
 temp$PHENO <- CANCER
 temp1[[CANCER]][[c("MALE","FEMALE")[SEX]]] <- temp

 temp1. <- rbind(temp1.,temp)

 }
}

OUTPUT.FILE = "AUC_common_cancer_scoresum"

temp1.$PGS.ID <- str_replace(temp1.$PRS,"SD_","")
temp1. <- merge(temp1.,common,by="PGS.ID")

tmp.out = paste0("output/",OUTPUT.FILE,"_quintiles_labeled_","20yr","_revision.csv")
print(tmp.out)
write.csv(temp1.,tmp.out,row.names = F)

### Quintile (1st ref)

auc.quintile.table <- function(DATA,COL.PHENO,COL.TIME,NO.YEAR=5,VAR.LIST,MEAN.SD){
 out = NULL
 DATA$PHENO_0 <- DATA[,COL.PHENO]
 DATA$TIME <- DATA[,COL.TIME]
 DATA <- DATA[DATA$PHENO_0%in%c(0,1) & !is.na(DATA$TIME),]
 DATA$PHENO_0[DATA$TIME >= NO.YEAR] <- 0
 DATA$TIME[DATA$TIME >= NO.YEAR] <- NO.YEAR

 for(SCORE in VAR.LIST){
 VAR1 <- DATA[,SCORE]
 mu <- MEAN.SD$Mean_control[MEAN.SD$PRS==SCORE]
 std <- MEAN.SD$SD_control[MEAN.SD$PRS==SCORE]
 VAR1. <- (VAR1-mu)/std
 DATA <- cbind(DATA,VAR1.)
 SD.VAR <- paste0("SD_",as.character(str_split(SCORE,"_")[[1]][2]))
 colnames(DATA)[ncol(DATA)] <- SD.VAR

 tmp <- rep(0,nrow(DATA))
 tmp[VAR1.>qnorm(.8)] <- "Q5"
 tmp[VAR1.>qnorm(.6) & VAR1.<=qnorm(.8)] <- "Q4"
 tmp[VAR1.>qnorm(.4) & VAR1.<=qnorm(.6)] <- "Q3"
 tmp[VAR1.>qnorm(.2) & VAR1.<=qnorm(.4)] <- "Q2"
 tmp[VAR1.<=qnorm(.2)] <- "Q1"


 tmp <- factor(tmp,paste0("Q",c(1,2,3,4,5)))

 tmp.con <- as.numeric(as.character(str_replace(tmp,"Q","")))

 DATA <- cbind(DATA,tmp,tmp.con) ## compare to 40-60
 PER.VAR <- paste0("PER_",as.character(str_split(SCORE,"_")[[1]][2]))
 CON.VAR <- paste0("CON_",as.character(str_split(SCORE,"_")[[1]][2]))
 colnames(DATA)[ncol(DATA)-1] <- PER.VAR
 colnames(DATA)[ncol(DATA)] <- CON.VAR
 print(table(DATA[DATA[,"PHENO_0"]==1,PER.VAR]))
 print(table(DATA[DATA[,"PHENO_0"]==1,CON.VAR]))

 cases <- table(DATA[DATA[,"PHENO_0"]==1,PER.VAR])


 # GLM for CI
 fit <- glm(formula(paste0("PHENO_0~",PER.VAR,"+age_recruitment")),data=DATA,family="binomial")
 temp <- roc(DATA$PHENO_0~predict(fit,DATA,type="response"))
 temp2 <- ci(temp)

 # Cox quintile
 cox <- coxph(formula(paste0("Surv(TIME,PHENO_0)~",PER.VAR,"+age_recruitment")),data=DATA)
 p.value <- summary(cox)$coefficients[,5]
 coeff <- summary(cox)$conf.int
 HR.cox <- coeff[,1]
 CI <- cbind(coeff[,3],coeff[,4])
 ph.pvalue <- cox.zph(cox)$table[1,3]

 ci. = character(nrow(CI))
 for(r in 1:nrow(CI)){
 ci.[r] <- form.ci1(HR.cox[r],CI[r,],dp=3)
 }
 tmp1 = NULL
 for(q in 1:length(ci.)){
 tmp1 <- c(tmp1,ci.[q],form.e(p.value[q]))
 }
 tmp1.names <- paste0(rep(c("HR (95%CI)","P (Cox HR)"),4),"_Q",kronecker(c(2,3,4,5),rep(1,2)))

 # p-trend
 cox.con <- coxph(formula(paste0("Surv(TIME,PHENO_0)~",CON.VAR,"+age_recruitment")),data=DATA)
 p.trend <- summary(cox.con)$coefficients[,5]

 temp.out <- as.data.frame(t(c(SD.VAR,form.ci1(temp2[2],temp2[c(1,3)],dp=3),
 tmp1,form.e(p.trend)[1],form.2(ph.pvalue,dp=3),
 form.2(mean(VAR1),dp=3),form.e(sd(VAR1),dp=3),
 nrow(DATA),
 temp2[c(2,1,3)],
 cases
 )))

 # output
 colnames(temp.out) <- c("PRS","AUC (95%CI)",
 tmp1.names,"age at recruitment","P (Cox HR)_age","Ptrend","P (PH assumption)",
 "Mean","SD","N",
 "auc","auc.lower","auc.upper",
 names(cases)
 )

 out <- rbind(out,temp.out)
 }
 return(as.data.frame(out))
}

mean.sd <- read.csv("data/Common_cancers_mean_sd_revision.csv")

temp1 = list()
temp1. = NULL
for(CANCER in cancer.list){
 for(SEX in sex.list[[CANCER]]){
 temp <- auc.quintile.table(DATA=data[data$V9_gender==SEX,],
 COL.PHENO=paste0("PHENO.",CANCER),
 COL.TIME=paste0("TIME.",CANCER),
 VAR.LIST=scores.list.[[CANCER]],
 MEAN.SD=mean.sd[mean.sd$Sex==c("MALE","FEMALE")[SEX],],
 NO.YEAR=20)
 temp$SEX <- c("MALE","FEMALE")[SEX]
 temp$PHENO <- CANCER
 temp1[[CANCER]][[c("MALE","FEMALE")[SEX]]] <- temp

 temp1. <- rbind(temp1.,temp)

 }
}

mean.sd <- read.csv("data/Common_cancers_mean_sd_revision.csv")

temp1 = list()
temp1. = NULL
for(CANCER in cancer.list){
 for(SEX in sex.list[[CANCER]]){
 temp <- auc.quintile.table(DATA=data[data$V9_gender==SEX,],
 COL.PHENO=paste0("PHENO.",CANCER),
 COL.TIME=paste0("TIME.",CANCER),
 VAR.LIST=scores.list.[[CANCER]],
 MEAN.SD=mean.sd[mean.sd$Sex==c("MALE","FEMALE")[SEX],],
 NO.YEAR=20)
 temp$SEX <- c("MALE","FEMALE")[SEX]
 temp$PHENO <- CANCER
 temp1[[CANCER]][[c("MALE","FEMALE")[SEX]]] <- temp

 temp1. <- rbind(temp1.,temp)

 }
}

OUTPUT.FILE = "AUC_common_cancer_scoresum"

temp1.$PGS.ID <- str_replace(temp1.$PRS,"SD_","")
temp1. <- merge(temp1.,common,by="PGS.ID")

tmp.out = paste0("output/",OUTPUT.FILE,"_quintiles_1st_labeled_","20yr","_revision.csv")
print(tmp.out)
write.csv(temp1.,tmp.out,row.names = F)

### Plot for Per SD

YEAR="20yr"
OUTPUT.FILE = "AUC_common_cancer_scoresum"
TYPE = "SD"

temp1 <- read.csv(paste0("output/",OUTPUT.FILE,"_",TYPE,"_labeled_",YEAR,"_revision.csv"))

dataplot <- temp1[,c("PGS.ID","AUC..95.CI.","HR..95.CI.","P..Cox.HR.","Number.valid.predictors","Number.of.Variants","simplified","simplified.type","simplified.ancestry","EastAsian.percent","N","SEX","auc","auc.lower","auc.upper")]


temp <- dataplot$HR..95.CI.
dataplot$HR <- as.numeric(sapply(temp,function(x){str_split(x," \\(")[[1]][1]}))
dataplot$HR.lower <- as.numeric(sapply(temp,function(x){x1 <- str_split(x,"\\(")[[1]][2]; str_split(x1," \226 ")[[1]][1]}))
dataplot$HR.upper <- as.numeric(sapply(temp,function(x){x1 <- str_split(x,"\226 ")[[1]][2]; str_replace(x1,"\\)","")}))

dataplot. <- dataplot[order(dataplot$auc),]
temp <- dataplot[!duplicated(dataplot$PGS.ID),c("PGS.ID","auc")]
temp <- temp[order(temp$auc),]
dataplot.$PGS.ID <- factor(dataplot.$PGS.ID,temp$PGS.ID )

temp1 <- cbind(dataplot.[, c("PGS.ID","Number.valid.predictors","Number.of.Variants","simplified","simplified.type","simplified.ancestry","EastAsian.percent","N","SEX","auc","auc.lower","auc.upper")],"AUC")

temp2 <- cbind(dataplot.[, c("PGS.ID","Number.valid.predictors","Number.of.Variants","simplified","simplified.type","simplified.ancestry","EastAsian.percent","N","SEX","HR","HR.lower","HR.upper")],"HR")

colnames(temp1) = colnames(temp2) = c("PGS.ID","Number.valid.predictors","Number.of.Variants","simplified","simplified.type","simplified.ancestry","EastAsian.percent","N","SEX","value","lower","upper","Type")

dataplot.1 = rbind(temp1,temp2)

dataplot.1$Number.of.Variants <- as.numeric(dataplot.1$Number.of.Variants)

dataplot.1$Number.of.Variants. <- log10(dataplot.1$Number.of.Variants)

CANCER = "Breast"
for(CANCER in c("Breast","Prostate")){
 p1 <- ggplot(data=dataplot.1[dataplot.1$simplified == CANCER & dataplot.1$Type=="AUC",],aes(x=PGS.ID,y=value,color=Number.of.Variants.)) +
 geom_hline(yintercept = .5,color="Grey",linetype="dashed") +
 geom_point() +
 geom_segment(aes(x=PGS.ID,y=lower,xend=PGS.ID,yend=upper,color=Number.of.Variants.)) +
 labs(y="AUC",color="Log10 (Number of variants in PGS)") +
 coord_cartesian(ylim=c(0.4,.75)) +
 scale_color_gradient(low = "blue",high="red") +
 theme_bw() +
 theme(legend.position = "top",
 axis.text.x = element_blank(),
 axis.ticks.x = element_blank(),
 axis.title.x = element_blank())
 p2 <- ggplot(data=dataplot.1[dataplot.1$simplified == CANCER & dataplot.1$Type=="HR",],aes(x=PGS.ID,y=value,color=Number.of.Variants.)) +
 geom_hline(yintercept = 1,color="Grey",linetype="dashed") +
 geom_point() +
 geom_segment(aes(x=PGS.ID,y=lower,xend=PGS.ID,yend=upper,color=Number.of.Variants.)) +
 labs(y="HR per SD change",color="Log10 (Number of variants in PGS)") +
 coord_cartesian(ylim=c(.75,2)) +
 scale_y_continuous(breaks = seq(.75,2,.25), labels = seq(.75,2,.25)) +
 scale_color_gradient(low = "blue",high="red") +
 theme_bw() +
 theme(axis.text.x = element_text(angle=90,hjust=0,vjust=.5),legend.position = "top")

 png(paste0("plot/AUC_",CANCER,"_",TYPE,"_",YEAR,"_revision.png"),res=180,width=2000,height=1000)
 print(ggarrange(p1,p2,nrow = 2,common.legend = T
 # labels = c("A) AUC","B) HR per standard deviation change"),label.x = 0.05,hjust = 0, label.y=.95,vjust=1,font.label=list(size=12,face="plain")
 ))
 dev.off()
}

CANCER = "Colorectal"

for(CANCER in c("Colorectal","Lung")){
 dataplot.1$SEX <- factor(dataplot.1$SEX,c("ALL","MALE","FEMALE"))
 p1 <- ggplot(data=dataplot.1[dataplot.1$simplified == CANCER & dataplot.1$Type=="AUC",],aes(x=PGS.ID,y=value,color=Number.of.Variants.)) +
 geom_hline(yintercept = .5,color="Grey",linetype="dashed") +
 geom_point(show.legend = F) +
 geom_segment(aes(x=PGS.ID,y=lower,xend=PGS.ID,yend=upper,color=Number.of.Variants.)) +
 labs(y="",color="") +
 coord_cartesian(ylim=c(0.475,.75)) +
 scale_y_continuous(breaks = seq(.45,.75,.05), labels = seq(.45,.75,.05)) +
 scale_color_gradient(low = "blue",high="red") +
 theme_bw() +
 theme(legend.position = "top",
 axis.text.x = element_blank(),
 axis.ticks.x = element_blank(),
 axis.title.x = element_blank())
 p2 <- ggplot(data=dataplot.1[dataplot.1$simplified == CANCER & dataplot.1$Type=="HR",],aes(x=PGS.ID,y=value,color=Number.of.Variants.)) +
 geom_hline(yintercept = 1,color="Grey",linetype="dashed") +
 geom_point() +
 geom_segment(aes(x=PGS.ID,y=lower,xend=PGS.ID,yend=upper,color=Number.of.Variants.)) +
 labs(y="",color="") +
 coord_cartesian(ylim=c(.75,2)) +
 scale_y_continuous(breaks = seq(.75,2,.25), labels = seq(.75,2,.25)) +
 scale_color_gradient(low = "blue",high="red") +
 theme_bw() +
 theme(axis.text.x = element_text(angle=90,hjust=0,vjust=.5),legend.position = "top")

 type.lab <- c("AUC","HR per SD")
 names(type.lab) <- c("AUC","HR")

 png(paste0("plot/AUC_",CANCER,"_",TYPE,"_",YEAR,"_revision.png"),res=180,width=1500,height=1200)
 print(ggarrange(p1+facet_grid(Type~SEX),
 p2+facet_grid(Type~SEX,labeller = labeller(Type=type.lab))+theme(strip.text.x =element_blank()),
 nrow = 2, common.legend = T, legend = "top"))
 dev.off()
}

## STABLE 3 and 4

YEAR="20yr"
INPUT.FILE = "AUC_common_cancer_scoresum"
OUTPUT.FILE = "sTable3_AUC"
TYPE = "SD"

temp1 <- read.csv(paste0("output/",INPUT.FILE,"_",TYPE,"_labeled_",YEAR,"_revision.csv"))
distribution <- read.csv(paste0("output/Distribution ttest_20yr","_revision.csv"))


stable <- temp1[,c("PGS.ID","simplified","SEX","Mean","SD","N",
 paste0("unadjusted.",c("AUC..95.CI.","HR..95.CI.","P..Cox.HR.","P..PH.assumption.")),
 "AUC..95.CI.","HR..95.CI.","P..Cox.HR.","P..PH.assumption.",
 "Number.of.Variants","Number.valid.predictors","Percentage.Missing.Predictors","simplified.type","auc")]

stable <- merge(stable,distribution[,!colnames(distribution)%in%c("PRS","PHENO")],by=c("PGS.ID","SEX"))

stable <- stable[,c("PGS.ID","simplified","SEX","Mean","SD","N","Ncase","Nnoncases",
 "MeanSD_case","MeanSD_noncase","X.MeanDifference_95.CI",
 "Pvalue_ttest",
 paste0("unadjusted.",c("AUC..95.CI.","HR..95.CI.","P..Cox.HR.","P..PH.assumption.")),
 "AUC..95.CI.","HR..95.CI.","P..Cox.HR.","P..PH.assumption.",
 "Number.of.Variants","Number.valid.predictors",
 "Percentage.Missing.Predictors","simplified.type","auc")]

stable <- stable[order(stable$auc,decreasing = T),]

stable <- stable[order(stable$PGS.ID),]
stable <- stable[order(stable$simplified),]

# Supplementary Table 3. Hazards ratios of per standard deviation increase of standardized polygenic risk scores (PRS), of cancer-specific PRSs.
write.csv(stable[,-c(ncol(stable))],paste0("output/",OUTPUT.FILE,"_",TYPE,"_labeled_",YEAR,"_revision.csv"),row.names = F)


YEAR="20yr"
INPUT.FILE = "AUC_common_cancer_scoresum"
OUTPUT.FILE = "sTable4_AUC"
TYPE = "quintiles_1st"
tmp1.names <- paste0(rep(c("HR..95.CI.","P..Cox.HR."),4),"_Q",kronecker(c(2,3,4,5),rep(1,2)))
tmp1.names2 <- paste0(rep(c("HR..95.CI.","P..Cox.HR."),3))
temp1 <- read.csv(paste0("output/",INPUT.FILE,"_",TYPE,"_labeled_",YEAR,"_revision.csv"))
stable <- temp1[,c("PGS.ID","simplified","SEX","Mean","SD","N","AUC..95.CI.",tmp1.names,
 "age.at.recruitment","P..Cox.HR._age","Ptrend","P..PH.assumption.","Number.of.Variants","Number.valid.predictors","Percentage.Missing.Predictors","simplified.type","auc")]
stable <- stable[order(stable$PGS.ID,decreasing = F),]

# Supplementary Table 4. Hazards ratios of the association between standardized polygenic risk scores (PRS) in quintiles and development of cancer.
write.csv(stable[,-c(ncol(stable))],paste0("output/",OUTPUT.FILE,"_",TYPE,"_labeled_",YEAR,"_revision.csv"),row.names = F)

# OUTPUT for main text

## Selection

YEAR="20yr"
INPUT.FILE = "AUC_common_cancer_scoresum"
OUTPUT.FILE = "Selection"
TYPE = "SD"
temp1 <- read.csv(paste0("output/",INPUT.FILE,"_",TYPE,"_labeled_",YEAR,"_revision.csv"))
stable <- temp1[,c("PGS.ID","simplified","SEX","Mean","SD","N","AUC..95.CI.","HR..95.CI.","P..Cox.HR.","P..PH.assumption.","Number.of.Variants","Number.valid.predictors","Percentage.Missing.Predictors","PGS.Development.Method","Type.of.Variant.Weight","simplified.type","auc")]

temp1$score <- paste0("SCORESUM_",temp1$PGS.ID)

select = NULL
for(CANCER in cancer.list){
 for(SEX in sex.list[[CANCER]]){
 temp <- temp1[temp1$simplified==CANCER & temp1$SEX==c("MALE","FEMALE")[SEX],]
 id <- which.max(temp$auc)
 select = rbind(select,temp[id,])
 }
}

write.csv(select,paste0("output/",OUTPUT.FILE,"_",TYPE,"_labeled_",YEAR,"_revision.csv"),row.names = F)

select[,c("PGS.ID","simplified","SEX","AUC..95.CI.")]

YEAR="20yr"
INPUT.FILE = "AUC_common_cancer_scoresum"
OUTPUT.FILE = "Selection"
TYPE = "SD"
mean.sd <- read.csv("data/Common_cancers_mean_sd_revision.csv")

temp1 <- read.csv(paste0("output/",OUTPUT.FILE,"_",TYPE,"_labeled_",YEAR,"_revision.csv"))

select.list <- temp1[,c("PGS.ID","simplified","SEX")]

data.s <- data[,c(which(!str_detect(colnames(data),"SCORESUM")),which(str_detect(colnames(data),paste0(select.list$PGS.ID,collapse = "|"))))]

for(i in 1:nrow(select.list)){
 MEAN.SD=mean.sd[mean.sd$Sex==select.list$SEX[i] & mean.sd$PGS.ID==select.list$PGS.ID[i],]
 mu <- MEAN.SD$Mean_control
 sd <- MEAN.SD$SD_control

 # SD
 SEX <- ifelse(select.list$SEX[i]=="MALE",1,2)
 id <- which(data.s$V9_gender==SEX)
 temp <- data.s[id,str_detect(colnames(data.s),select.list$PGS.ID[i])]
 temp <- (temp-mu)/sd

 temp.out <- rep(NA,nrow(data.s))
 temp.out[id] <- temp
 data.s <- cbind(data.s,temp.out)
 SD.VAR <- paste0("SD_",select.list$PGS.ID[i])
 colnames(data.s)[ncol(data.s)] <- SD.VAR

 # Quintile
 tmp <- rep(0,length(temp))
 tmp[temp>qnorm(.8)] <- "Q5"
 tmp[temp>qnorm(.6) & temp<=qnorm(.8)] <- "Q4"
 tmp[temp>qnorm(.4) & temp<=qnorm(.6)] <- "Q3"
 tmp[temp>qnorm(.2) & temp<=qnorm(.4)] <- "Q2"
 tmp[temp<=qnorm(.2)] <- "Q1"

 temp.out <- rep(NA,nrow(data.s))
 temp.out[id] <- tmp
 data.s <- cbind(data.s,temp.out)
 Q.VAR <- paste0("Q_",select.list$PGS.ID[i])
 colnames(data.s)[ncol(data.s)] <- Q.VAR
}

## FIGURE 1

### Plots distribution and ROC (Discrimination)

font.size=3
font.size.axis = 10

data.d <- read.csv(paste0("output/Distribution ttest_20yr","_revision.csv"))

dataplot.d = lab.d = list()
for(CANCER in cancer.list){
 for(SEX in sex.list[[CANCER]]){
 temp <- select.list[select.list$simplified==CANCER & select.list$SEX==c("MALE","FEMALE")[SEX],]
 temp.p <- data.s[data.s$V9_gender==SEX,c(which(colnames(data.s)==paste0("SD_",temp$PGS.ID)),
 which(colnames(data.s)==paste0("PHENO.",CANCER)),
 which(colnames(data.s)==paste0("TIME.",CANCER)),
 which(colnames(data.s)=="age_recruitment"))]
 temp.p$PGS.ID <- temp$PGS.ID
 colnames(temp.p) <- c("Standardised_PRS","pheno","time","age_recruitment","PGS.ID")

 dataplot.d[[CANCER]][[c("MALE","FEMALE")[SEX]]] <- temp.p


 tmp <- data.d[data.d$PGS.ID==temp$PGS.ID & data.d$SEX==temp$SEX,c("X.MeanDifference_95.CI","Pvalue_ttest")]

 temp.d <- as.data.frame(t(c(paste0("t-test, p-value=",form.e(as.numeric(tmp[2]),dp=2)),
 0.5,0)))

 colnames(temp.d) <- c("Text","y","x")

 lab.d[[CANCER]][[c("MALE","FEMALE")[SEX]]] <- temp.d
 }
}

# distribution
plot.d = list()
for(CANCER in cancer.list){
 for(SEX in sex.list[[CANCER]]){

 plot.d[[CANCER]][[c("MALE","FEMALE")[SEX]]] <- ggplot(data=dataplot.d[[CANCER]][[c("MALE","FEMALE")[SEX]]],aes(x=Standardised_PRS,color=factor(pheno))) +
 geom_vline(xintercept = 0, color="grey") +
 geom_vline(xintercept = 2.5, color="grey") +
 geom_vline(xintercept = -2.5, color="grey") +
 geom_density(show.legend = F) +
 scale_color_manual(breaks=c(0,1),values=c("black","red"),labels=c("Non-cases","Cases")) +
 labs(x= "Standardised PRS",y="",color="") +
 geom_text(x=2,y=0.3,label="Cases",color="red",size=font.size,hjust=0) +
 geom_text(x=-1.35,y=0.27,label="Non-cases",color="black",size=font.size,hjust=1) +
 geom_text(data=lab.d[[CANCER]][[c("MALE","FEMALE")[SEX]]],aes(x=as.numeric(x),y=as.numeric(y),label=Text),color="black",size=font.size,vjust=1) +
 coord_cartesian(ylim=c(0,.5),xlim=c(-3.5,3.5)) +
 scale_x_continuous(breaks = c(-2.5,0,2.5), labels = c(-2.5,0,2.5)) +
 theme_half_open() +
 theme(axis.text.y = element_blank(),axis.ticks.y = element_blank(),axis.line.y = element_blank(),
 axis.title.x = element_text(size=font.size.axis),axis.text=element_text(size=font.size.axis))

 }
}

distribution <- ggarrange(plot.d[["Breast"]][["FEMALE"]],
 plot.d[["Colorectal"]][["MALE"]],plot.d[["Colorectal"]][["FEMALE"]],
 plot.d[["Lung"]][["MALE"]],plot.d[["Lung"]][["FEMALE"]],
 plot.d[["Prostate"]][["MALE"]],nrow=1)


# ROC


data.a <- read.csv(paste0("output/AUC_common_cancer_scoresum_SD_labeled_20yr_revision.csv"))

for(CANCER in cancer.list){
 for(SEX in sex.list[[CANCER]]){
 DATA = data.a[data.a$SEX==c("MALE","FEMALE")[SEX] & data.a$simplified==CANCER,]
 fit <- lm(auc~Number.of.Variants,data=DATA)
 print(paste0(CANCER," \226 ",c("male","female")[SEX]))
 print(summary(fit))
 }
}
dataplot.r = list()
dataplot.r.ci = list()
NO.YEAR = 20
for(CANCER in cancer.list){
 for(SEX in sex.list[[CANCER]]){

 DATA = dataplot.d[[CANCER]][[c("MALE","FEMALE")[SEX]]]
 DATA$PHENO_0 <- DATA[,"pheno"]
 DATA$TIME <- DATA[,"time"]
 DATA <- DATA[DATA$PHENO_0%in%c(0,1) & !is.na(DATA$TIME),]
 DATA$PHENO_0[DATA$TIME >= NO.YEAR] <- 0
 DATA$TIME[DATA$TIME >= NO.YEAR] <- NO.YEAR

 fit.u <- glm(formula(paste0("PHENO_0~Standardised_PRS")),data=DATA)
 temp.u <- roc(DATA$PHENO_0~predict(fit.u,DATA,type="response"))

 temp.r.u <- as.data.frame(cbind("Unadjusted",temp.u$sensitivities,temp.u$specificities))
 colnames(temp.r.u)<- c("Type","Sensitivity","Specificity")
 tmp.u. <- ci(temp.u)
 tmp.u <- form.ci1(tmp.u.[2],tmp.u.[c(1,3)],dp=2)
 tmp.u <- str_replace_all(tmp.u, "\\\x96","-")

 fit <- glm(formula(paste0("PHENO_0~Standardised_PRS+age_recruitment")),data=DATA)
 temp <- roc(DATA$PHENO_0~predict(fit,DATA,type="response"))

 temp.r <- as.data.frame(cbind("Adjusted",temp$sensitivities,temp$specificities))
 colnames(temp.r)<- c("Type","Sensitivity","Specificity")

 dataplot.r[[CANCER]][[c("MALE","FEMALE")[SEX]]] <- rbind(temp.r.u,temp.r)

 temp <- select.list[select.list$simplified==CANCER & select.list$SEX==c("MALE","FEMALE")[SEX],]
 tmp <- data.a[data.a$PGS.ID==temp$PGS.ID & data.a$SEX==temp$SEX,c("AUC..95.CI.")]

 tmp <- str_replace_all(tmp, "\\\x96","-")
 dataplot.r.ci[[CANCER]][[c("MALE","FEMALE")[SEX]]] <- paste0("i) ",tmp.u,"\n","ii) ",tmp)
 }
}
axis.roc = seq(0,1,.2)
plot.r = list()
for(CANCER in cancer.list){
 for(SEX in sex.list[[CANCER]]){
 plot.r[[CANCER]][[c("MALE","FEMALE")[SEX]]] <- ggplot(data=dataplot.r[[CANCER]][[c("MALE","FEMALE")[SEX]]],aes(x=as.numeric(Specificity),y=as.numeric(Sensitivity),color=Type)) +
 geom_abline(intercept = 1,slope = 1, color="grey",linetype="dashed") +
 geom_line(aes(linetype=Type),show.legend = F) +
 scale_linetype_manual(breaks=c("Unadjusted","Adjusted"),
 values=c("solid","dashed")) +
 scale_color_manual(breaks=c("Unadjusted","Adjusted"),
 values=c("black","grey")) +
 scale_y_continuous(labels =axis.roc, breaks = axis.roc) +
 scale_x_reverse(labels =axis.roc, breaks = axis.roc) +
 labs(x="Specificity",y="Sensitivity") +
 geom_text(x=0,y=.1,label=dataplot.r.ci[[CANCER]][[c("MALE","FEMALE")[SEX]]],hjust=1,size=font.size,show.legend = F,color="black") +
 # scale_x_continuous(labels =axis.roc, breaks = axis.roc) +
 theme_half_open() +
 theme(axis.title = element_text(size=font.size.axis),axis.text=element_text(size=font.size.axis))
 }
}

roc.curves <- ggarrange(plot.r[["Breast"]][["FEMALE"]],
 plot.r[["Prostate"]][["MALE"]],
 plot.r[["Colorectal"]][["FEMALE"]],
 plot.r[["Colorectal"]][["MALE"]],
 plot.r[["Lung"]][["FEMALE"]],
 plot.r[["Lung"]][["MALE"]],
 nrow=1)

### Plot absolute risk curves

ETHNICITY = "CHINESE"
# select 1%,every 5%, 99%
select.column <- seq(0,100,5)
select.column[1] <- 1
select.column[length(select.column)] <- 99
age.col = "age"
select.column <- select.column[-c(1,length(select.column))]

plot.a <- list()
for(CANCER in cancer.list){
 for(SEX in sex.list[[CANCER]]){
 PGS = select.list$PGS.ID[select.list$simplified==CANCER & select.list$SEX==c("MALE","FEMALE")[SEX]]
 SCORE = paste0("SCORESUM_",PGS)

 out.file.name = paste0("data/absolute_risk_tables_",CANCER,"_revision/")
 absolute.risk.table <-
 readRDS(paste0(out.file.name,SCORE,"_",c("MALE","FEMALE")[SEX],"_",ETHNICITY,"_5yr.rds"))

 AR5_g <- absolute.risk.table[,c(1,which(colnames(absolute.risk.table)%in%select.column))]
 AR5_g <- as.data.frame(AR5_g[20:75,])
 melted.arg.5 <- melt(AR5_g, id = "AGE")
 colour <- hue_pal()(length(select.column))

 melted.arg.5$value <- as.numeric(melted.arg.5$value)
 upper.limit = 0.08

 p <- ggplot(melted.arg.5, aes(x = AGE, y= value, color = variable))+
 geom_line(data=melted.arg.5, aes(x = AGE, y= value, color = variable),show.legend = F) +
 scale_color_manual(breaks=levels(factor(melted.arg.5$variable)),
 values = colour,
 # guide=guide_legend(override.aes = list(color=colour)),
 # name=bquote("PRS percentiles")
 ) +
 labs(x="Age, years",y="Five-year absolute risk (%)") +
 scale_x_continuous(labels = c(seq(20, 70,10)),breaks= c(seq(20, 70,10)), expand = c(0, 0)) +
 guides(color=guide_legend(nrow=1,direction="horizontal")) +
 coord_cartesian(xlim=c(23,77),ylim = c(0,upper.limit)) +
 scale_y_continuous(labels =format(round(seq(0,upper.limit,.01)*100,0)), breaks = seq(0,upper.limit,.01)) +
 theme_half_open() +
 theme(axis.title = element_text(size=font.size.axis),axis.text=element_text(size=font.size.axis),
 # legend.position="bottom",panel.grid.minor=element_blank(),legend.text=element_text(size=font.size.axis),legend.title=element_text(size=font.size.axis),
 # legend.margin=margin(-10, 0, 0, 0),
 plot.margin=margin(0,2,6,15))


 plot.a[[CANCER]][[c("MALE","FEMALE")[SEX]]] <- p
 }
}


abs.curves <- ggarrange(plot.a[["Breast"]][["FEMALE"]],
 plot.a[["Prostate"]][["MALE"]],
 plot.a[["Colorectal"]][["FEMALE"]],
 plot.a[["Colorectal"]][["MALE"]],
 plot.a[["Lung"]][["FEMALE"]],
 plot.a[["Lung"]][["MALE"]],
 nrow=1,common.legend = T)

### Plot by decile (Calibration)

Estimates and 95% CI of the calibration slope and intercept are reported based on a linear regression of the decile-specific observed proportion of cases within 5 years and the average of the predicted 5-year absolute risk.

axis =c(0,3.5)
plot.c = list()

for(CANCER in cancer.list){
 for(SEX in sex.list[[CANCER]]){
 PGS = select.list$PGS.ID[select.list$simplified==CANCER & select.list$SEX==c("MALE","FEMALE")[SEX]]
 SCORE = paste0("SCORESUM_",PGS)

 cal.data. <- read.csv(paste0("output/Calibration_",CANCER,"_",c("MALE","FEMALE")[SEX],"_5yr_revision.csv"))
 dataplot <- as.data.frame(cal.data.[cal.data.$Order!=0 & cal.data.$PGS==SCORE ,c("PGS","Decile","Order","Observed","Expected","N")])
 dataplot$Order <- factor(dataplot$Order,1:10)
 for(i in 4:6){
 dataplot[,i] <- as.numeric(as.character(dataplot[,i]))
 }

 dataplot$proportion <- dataplot$Observed/dataplot$N

 dataplot$upper = dataplot$lower = rep(NA,nrow(dataplot))
 for(i in 1:nrow(dataplot)){
 dataplot[i,c("lower","upper")] <- as.numeric(prop.test(dataplot$Observed[i],dataplot$N[i],correct=FALSE)$conf.int[1:2])
 }

 dataplot$proportion.o <-dataplot$proportion * 100
 dataplot$upper <- dataplot$upper *100
 dataplot$lower <- dataplot$lower *100
 dataplot$proportion.e <- dataplot$Expected/dataplot$N *100

 fit <- lm(proportion.o~proportion.e,data=dataplot)
 coef <- as.data.frame(summary(fit)$coef[,1:2])
 coef$lower <- coef$Estimate - qnorm(.975) * coef$Estimate
 coef$upper <- coef$Estimate + qnorm(.975) * coef$Estimate
 slope.inter <- as.data.frame(cbind(SCORE,paste0(c("Intercept=","Slope="),
 form.2(coef$Estimate,dp=2),
 " (",form.2(coef$lower,dp=2),", ",
 form.2(coef$upper,dp=2),")")))
 colnames(slope.inter) <- c("PGS","Text")

 slope.inter$y <- c(3,3.4)
 slope.inter$x <- c(axis[2],axis[2])

 # Hosmer-Lemeshow test
 p <- paste0("Hosmer-Lemeshow P=",form.2(cal.data.[cal.data.$Order==0 & cal.data.$PGS==SCORE ,c("HL.p")],3))
 slope.inter <- rbind(slope.inter,c(SCORE,p,2.6,3.5))

 plot.c[[CANCER]][[c("MALE","FEMALE")[SEX]]] <- ggplot(data=dataplot, aes(x=as.numeric(proportion.e),y=as.numeric(proportion.o))) +
 geom_abline(intercept = 0, slope = 1,color="Grey",linetype="dashed") +
 geom_point(show.legend = F) +
 geom_segment(aes(x=as.numeric(proportion.e),xend=as.numeric(proportion.e),y=lower,yend=upper),show.legend = F) +
 geom_text(data=slope.inter,aes(x=as.numeric(x),y=as.numeric(y),label=Text),color="black",hjust=1,size=font.size) +
 coord_cartesian(xlim = axis,ylim=axis) +
 labs(x="Expected (%)",y="Observed (%)") +
 theme_half_open() +
 theme(axis.title=element_text(size=font.size.axis),axis.text=element_text(size=font.size.axis),
 plot.margin=margin(0,2,4,15))

 }
}

cal.curves <- ggarrange(plot.c[["Breast"]][["FEMALE"]],
 plot.c[["Prostate"]][["MALE"]],
 plot.c[["Colorectal"]][["FEMALE"]],
 plot.c[["Colorectal"]][["MALE"]],
 plot.c[["Lung"]][["FEMALE"]],
 plot.c[["Lung"]][["MALE"]],
 nrow=1,common.legend = T,legend = "bottom")

plot.label = list()

for(CANCER in cancer.list){
 for(SEX in sex.list[[CANCER]]){
 PGS = select.list$PGS.ID[select.list$simplified==CANCER & select.list$SEX==c("MALE","FEMALE")[SEX]]
 PGS.label = paste0(CANCER, " \226 ",c("male","female")[SEX],"\n",PGS)
 dataplot <- as.data.frame(list("text" = PGS.label,"x" = 0.5,"y" = 0.5))

 plot.label[[CANCER]][[c("MALE","FEMALE")[SEX]]] <- ggplot(data=dataplot,aes(x=x,y=y)) +
 geom_text(aes(label=text),fontface="bold") +
 coord_cartesian(ylim=c(.4,.6)) +
 theme_void()
 }
}

label.curves <- ggarrange(plot.label[["Breast"]][["FEMALE"]],
 plot.label[["Prostate"]][["MALE"]],
 plot.label[["Colorectal"]][["FEMALE"]],
 plot.label[["Colorectal"]][["MALE"]],
 plot.label[["Lung"]][["FEMALE"]],
 plot.label[["Lung"]][["MALE"]],
 nrow=1)

plot. <- ggarrange(distribution,roc.curves,abs.curves,cal.curves,nrow=4,labels = "AUTO",label.y = 1.05,label.x = 0,hjust = 0)

png(paste0("plot/Figure1.png"),res=300,width=4000,height=2500)
ggarrange(label.curves,plot.,nrow=2,heights = c(1,16))
dev.off()

tiff(paste0("plot/Figure1.tif"),res=600,width=7800,height=4500,compression="lzw")
ggarrange(label.curves,plot.,nrow=2,heights = c(1,16))
dev.off()

## TABLE

### Table 1

med <- function(VAR,DATA,dp=0){
 x = DATA[,VAR]
 temp <- summary(as.numeric(as.character(x)))
 print(temp)
 output <- c("Median",paste0(form.2(temp[3],dp)," (",form.2(temp[2],dp)," \226 ",form.2(temp[5],dp),")"))
 output <- rbind("",output)
 output <- cbind(VAR,output)
 colnames(output) <- c("VAR1","Levels","N")
 return(output)
}
range_edit <- function(x,dp=0){
 temp <- summary(as.numeric(as.character(x)))
 paste0(" (",form.2(temp[1],dp)," \226 ",form.2(temp[6],dp),")")
}
mean.sd <- function(x,dp=0){
 x <- as.numeric(as.character(x))
 paste0(form.2(mean(x),dp)," (",form.2(sd(x),dp),")")
}

tab1 <- function(VAR1,DATA,DP=0){
 var1 <- DATA[,VAR1]

 temp <- table(var1,useNA="ifany")
 temp1 <- temp/sum(temp)*100
 output <- cbind(names(temp),paste0(form(temp)," (",form.2(temp1,DP),")"))
 output <- rbind("",output)
 output <- cbind(VAR1,output)
 colnames(output) <- c("VAR1","Levels","N")
 return(output)
}

DATA= data.s
VAR1= "V9_gender"

age.list = list("Breast" = "age_breast",
 "Colorectal" = "age_crc",
 "Lung" = "age_lung",
 "Prostate" = "age_prostate")

lenfy.list = list("Breast" = "lenfy_breast",
 "Colorectal" = "lenfy_crc",
 "Lung" = "lenfy_lung",
 "Prostate" = "lenfy_prostate")

for(CANCER in cancer.list){
 id<- which(data.s$age_recruitment > data.s[,age.list[[CANCER]]] * !is.na(data.s[,age.list[[CANCER]]]))
 if(length(id)==0) print("No prevalent cases")
 if(length(id)!=0) print(id)
}


temp.agedx <- data.s[,colnames(data.s)[str_detect(colnames(data.s),"age") & !str_detect(colnames(data.s),"recruitment")]]
temp.min <- min(temp.agedx)

data.s$min.agedx <- NA

for(ind in 1:nrow(data.s)){
 temp <- temp.agedx[ind,]
 temp1 <- length(which(!is.na(temp)))
 if(temp1 >0){
 data.s$min.agedx[ind] <- min(temp,na.rm=T)
 }
}

temp.lenfy <- data.s[,colnames(data.s)[str_detect(colnames(data.s),"lenfy")]]
temp.max <- max.col(temp.lenfy)

data.s$max.lenfy <- 0
for(ind in 1:nrow(data.s)){
 data.s$max.lenfy[ind] <- temp.lenfy[ind,max.col(temp.lenfy)[ind]]
}


data.s$cancer <- rowSums(data.s[,colnames(data.s)[str_detect(colnames(data.s),"PHENO.")]])


var1.list <- c("V9_gender","cancer","V15","D5_2_2","D14_1","D14_5_3","D37_1_2","D51_3","D51_4","D67","D69","D70","D71")

sup.list <- c("D52_1_1","D52_3_1","D52_14","D52_16_1","D52_18","D52_6_1")


tab1.output = tab1.output. = list()
for(VAR1 in var1.list){
 temp <- as.data.frame(tab1(VAR1=VAR1,DATA=data.s))
 temp$merge.id <- paste0(temp$VAR1,temp$Levels)
 tab1.output[["ALL"]][["ALL"]][[VAR1]] <- temp
}

for(SEX in c(1,2)){
 for(VAR1 in var1.list){
 temp <- as.data.frame(tab1(VAR1=VAR1,DATA=data.s[data.s$V9_gender==SEX,]))
 temp$merge.id <- paste0(temp$VAR1,temp$Levels)
 tab1.output[["ALL"]][[c("MALE","FEMALE")[SEX]]][[VAR1]] <- temp
 }
}

for(CANCER in cancer.list){
 for(SEX in sex.list[[CANCER]]){
 for(VAR1 in var1.list){
 temp <- as.data.frame(tab1(VAR1=VAR1,DATA=data.s[data.s[,paste0("PHENO.",CANCER)]==1 & data.s$V9_gender==SEX,]))
 temp$merge.id <- paste0(temp$VAR1,temp$Levels)
 tab1.output[[CANCER]][[c("MALE","FEMALE")[SEX]]][[VAR1]] <- temp

 }
 tab1.output.[[CANCER]][[c("MALE","FEMALE")[SEX]]] <- as.data.frame(do.call(rbind,tab1.output[[CANCER]]))
 }
}

var2.list <- c("age_recruitment","min.agedx","max.lenfy","D2")

for(VAR1 in var2.list){
 tmp <- as.data.frame(med(VAR=VAR1,DATA=data.s))
 tmp$merge.id <- paste0(tmp$VAR1,tmp$Levels)
 tab1.output[["ALL"]][["ALL"]][[VAR1]] <- tmp
}

for(SEX in c(1,2)){
 for(VAR1 in var2.list){
 tmp <- as.data.frame(med(VAR=VAR1,DATA=data.s[data.s$V9_gender==SEX,]))
 tmp$merge.id <- paste0(tmp$VAR1,tmp$Levels)
 tab1.output[["ALL"]][[c("MALE","FEMALE")[SEX]]][[VAR1]] <- tmp
 }
}

for(CANCER in cancer.list){
 for(SEX in sex.list[[CANCER]]){
 for(VAR1 in var2.list[c(1,4)]){
 tmp <- as.data.frame(med(VAR=VAR1,DATA=data.s[data.s[,paste0("PHENO.",CANCER)]==1 & data.s$V9_gender==SEX,]))
 tmp$merge.id <- paste0(tmp$VAR1,tmp$Levels)
 tab1.output[[CANCER]][[c("MALE","FEMALE")[SEX]]][[VAR1]] <- tmp
 }
 }
}

for(CANCER in cancer.list){
 for(SEX in sex.list[[CANCER]]){
 tmp <- as.data.frame(med(VAR=age.list[[CANCER]],DATA=data.s[data.s[,paste0("PHENO.",CANCER)]==1 & data.s$V9_gender==SEX,]))
 tmp$VAR1 <- "min.agedx"
 tmp$merge.id <- paste0(tmp$VAR1,tmp$Levels)
 tab1.output[[CANCER]][[c("MALE","FEMALE")[SEX]]][["min.agedx"]] <- tmp

 tmp <- as.data.frame(med(VAR=lenfy.list[[CANCER]],DATA=data.s[data.s[,paste0("PHENO.",CANCER)]==1 & data.s$V9_gender==SEX,]))
 tmp$VAR1 <- "max.lenfy"
 tmp$merge.id <- paste0(tmp$VAR1,tmp$Levels)
 tab1.output[[CANCER]][[c("MALE","FEMALE")[SEX]]][["max.lenfy"]] <- tmp
 }
}

order.list <- c("V9_gender","age_recruitment","cancer","min.agedx","max.lenfy","V15","D5_2_2","D2","D14_1","D14_5_3","D37_1_2","D51_3","D51_4","D67","D70","D69","D71")


temp.out = list()
CANCER = "ALL"
temp = NULL
for(ORD in order.list){
 temp <- rbind(temp,tab1.output[[CANCER]][["ALL"]][[ORD]])
}
temp.out[[CANCER]][["ALL"]] <- temp

for(SEX in c(1,2)){
 temp = NULL
 for(ORD in order.list){
 temp <- rbind(temp,tab1.output[[CANCER]][[c("MALE","FEMALE")[SEX]]][[ORD]])
 }
 temp.out[[CANCER]][[c("MALE","FEMALE")[SEX]]] <- temp
}

for(CANCER in c(cancer.list)){
 for(SEX in sex.list[[CANCER]]){
 temp = NULL
 for(ORD in order.list){
 temp <- rbind(temp,tab1.output[[CANCER]][[c("MALE","FEMALE")[SEX]]][[ORD]])
 }
 temp.out[[CANCER]][[c("MALE","FEMALE")[SEX]]] <- temp
 }
}

CANCER = "ALL"
temp = temp.out[[CANCER]][["ALL"]]
temp = cbind(1:nrow(temp),temp)
colnames(temp)[1] <- "order"
for(SEX in c(2,1)){
 temp2 <- temp.out[[CANCER]][[c("MALE","FEMALE")[SEX]]][,c("merge.id","N")]
 colnames(temp2) <- c("merge.id",paste0("N_",CANCER,"_",c("MALE","FEMALE")[SEX]))
 temp <- merge(temp,temp2,by="merge.id",all = T)
}

for(CANCER in cancer.list){
 for(SEX in sex.list[[CANCER]]){
 temp2 <- temp.out[[CANCER]][[c("MALE","FEMALE")[SEX]]][,c("merge.id","N")]
 colnames(temp2) <- c("merge.id",paste0("N_",CANCER,"_",c("MALE","FEMALE")[SEX]))
 temp <- merge(temp,temp2,by="merge.id",all = T)
 }
}
table1 <- temp[order(temp$order),]

write.csv(table1,"output/Table 1.csv",row.names=F)

### Table 2

OUTPUT.FILE = "AUC_common_cancer_scoresum"
tmp.out = paste0("output/",OUTPUT.FILE,"_quintiles_labeled_","20yr","_revision.csv")

quin <- read.csv(tmp.out)
temp.out = NULL
for(CANCER in cancer.list){
 for(SEX in sex.list[[CANCER]]){
 PGS = select.list$PGS.ID[select.list$simplified==CANCER & select.list$SEX==c("MALE","FEMALE")[SEX]]
 SCORE = paste0("SCORESUM_",PGS)
 temp <- quin[quin$PGS.ID ==PGS & quin$SEX==c("MALE","FEMALE")[SEX],]

 row0 <- as.data.frame(t(c(paste0(CANCER," \226 ",c("Male","Female")[SEX]),rep("",6))))
 row1 <- as.data.frame(t(c("Number of cases",temp[,paste0("Q",1:5)],"")))
 row2 <- as.data.frame(t(c("HR (95%CI)",temp[,paste0("HR..95.CI._Q",1:2)],"1.00 (Referent)",temp[,paste0("HR..95.CI._Q",4:5)],form.e(temp[,"Ptrend"],2))))

 colnames(row0) = colnames(row1) = colnames(row2) = c("Cancer site",paste0("Q",1:5),"P-trend")

 temp.out <- rbind(temp.out,row0,row1,row2)

 print(temp[,"P..PH.assumption."])

 }
}

write.csv(as.matrix(temp.out),"output/Table 2.csv",row.names = F)

### Table 3 - Adjustment

D2 Num 8 BMI(Kg/M**2) D67 Num 8 Cancer of 1st deg relatives D14_1 Num 8 Cigaret:No,Ex,Curr D37_1_2 Num 8 Alcohol:N/occ,Wkly,Daily V15 Num 4 FATHER DIALECT D51_3 Num 8 Moderat activ:No,1/2-3,4+hrs/week D51_4 Num 8 Wkly Vig wrk/Sren sports:No,Yes D5_2_2 Num 8 Educ:No,Prim,Sec+

table(data.s$D14_1,data.s$PHENO.Lung,data.s$V9_gender)

NO.YEAR = 20
out = list()
out.table = NULL
for(CANCER in cancer.list){
 for(SEX in sex.list[[CANCER]]){

 PGS = select.list$PGS.ID[select.list$simplified==CANCER & select.list$SEX==c("MALE","FEMALE")[SEX]]
 SD.VAR = paste0("SD_",PGS)
 Q.VAR = paste0("Q_",PGS)
 COL.PHENO=paste0("PHENO.",CANCER)
 COL.TIME=paste0("TIME.",CANCER)

 # print(PGS)

 DATA = data.s[data.s$V9_gender==SEX,]

 DATA$PHENO_0 <- DATA[,COL.PHENO]
 DATA$TIME <- DATA[,COL.TIME]
 DATA <- DATA[DATA$PHENO_0%in%c(0,1) & !is.na(DATA$TIME),]
 DATA$PHENO_0[DATA$TIME >= NO.YEAR] <- 0
 DATA$TIME[DATA$TIME >= NO.YEAR] <- NO.YEAR
 print(length(DATA$FID[DATA$PHENO_0==1] ))

 # Cox per SD
 cox <- coxph(formula(paste0("Surv(TIME,PHENO_0)~",SD.VAR,"+age_recruitment + as.factor(V15) + as.factor(D5_2_2) + D2 + as.factor(D14_1) + as.factor(D37_1_2) + as.factor(D51_3) + as.factor(D51_4) + as.factor(D67)")),data=DATA)
 # print(CANCER)
 # print(summary(cox))
 p.value <- summary(cox)$coefficients[,5]
 coeff <- summary(cox)$conf.int
 HR.cox <- coeff[,1]
 CI <- cbind(coeff[,3],coeff[,4])
 ph.pvalue <- cox.zph(cox)$table[1,3]

 ci.out = cbind(row.names(coeff),form.ci(HR.cox,CI,dp=2),form.e(p.value))
 colnames(ci.out) = paste0(c("VAR","HR","P"),"_",CANCER,"_",tolower(c("MALE","FEMALE"))[SEX])

 out[[CANCER]][[c("MALE","FEMALE")[SEX]]]<- ci.out
 out.table <- cbind(out.table,ci.out)

 print(paste0(CANCER," - ",SEX))
 print(cox.zph(cox)$table)
 }
}

write.csv(out.table,"output/Table 3.csv",row.names = F)

# Additional analysis

## Time-to-event metric (AUCs) at 5-year

surv.auc.table <- function(DATA,COL.PHENO,COL.TIME,NO.YEAR=5,VAR.LIST,MEAN.SD,HR.LIST){
 out = NULL
 DATA$PHENO_0 <- DATA[,COL.PHENO]
 DATA$TIME <- DATA[,COL.TIME]
 DATA <- DATA[DATA$PHENO_0%in%c(0,1) & !is.na(DATA$TIME),]
 DATA$PHENO_0[DATA$TIME >= NO.YEAR] <- 0
 DATA$TIME[DATA$TIME >= NO.YEAR] <- NO.YEAR

 for(SCORE in VAR.LIST){
 VAR1 <- DATA[,SCORE]
 mu <- MEAN.SD$Mean_control[MEAN.SD$PRS==SCORE]
 std <- MEAN.SD$SD_control[MEAN.SD$PRS==SCORE]
 VAR1. <- (VAR1-mu)/std
 DATA <- cbind(DATA,VAR1.)
 SD.VAR <- paste0("SD_",as.character(str_split(SCORE,"_")[[1]][2]))
 colnames(DATA)[ncol(DATA)] <- SD.VAR

 # Cox per SD
 cox <- coxph(formula(paste0("Surv(TIME,PHENO_0)~",SD.VAR,"+age_recruitment")),data=DATA)
 p.value <- summary(cox)$coefficients[1,5]
 coeff <- summary(cox)$conf.int
 HR.cox <- coeff[1,1]
 CI <- c(coeff[1,3],coeff[1,4])
 ph.pvalue <- cox.zph(cox)$table[1,3]

 SURV.AUC5 <- AUC.cd(Surv.rsp=Surv(DATA$TIME,DATA$PHENO_0), lp=predict(cox),lpnew=predict(cox), times=5)$auc

 # output
 temp.out <- as.data.frame(t(c(SD.VAR,SURV.AUC5)))

 colnames(temp.out) <- c("PRS","AUC_5yrs")
 out <- rbind(out,temp.out)
 }
 return(as.data.frame(out))
}

mean.sd <- read.csv("data/Common_cancers_mean_sd_revision.csv")


temp1 = list()
temp1. = NULL
for(CANCER in cancer.list){
 for(SEX in sex.list[[CANCER]]){
 temp <- surv.auc.table(DATA=data[data$V9_gender==SEX,],
 COL.PHENO=paste0("PHENO.",CANCER),
 COL.TIME=paste0("TIME.",CANCER),
 VAR.LIST=scores.list.[[CANCER]],
 MEAN.SD=mean.sd[mean.sd$Sex==c("MALE","FEMALE")[SEX],],
 NO.YEAR=20)
 temp$SEX <- c("MALE","FEMALE")[SEX]
 temp$PHENO <- CANCER
 temp1[[CANCER]][[c("MALE","FEMALE")[SEX]]] <- temp

 temp1. <- rbind(temp1.,temp)

 }
}

OUTPUT.FILE = "survAUC_common_cancer_scoresum"

temp1.$PGS.ID <- str_replace(temp1.$PRS,"SD_","")

temp1. <- merge(temp1.,common,by="PGS.ID")

write.csv(temp1.[,c("PGS.ID","simplified","SEX","AUC_5yrs")],paste0("output/sT5_",OUTPUT.FILE,"additional_analysis_revision.csv"),row.names = F)


temp1$score <- paste0("SCORESUM_",temp1$PGS.ID)

temp1 <- temp1.
select = NULL
for(CANCER in cancer.list){
 for(SEX in sex.list[[CANCER]]){
 temp <- temp1[temp1$simplified==CANCER & temp1$SEX==c("MALE","FEMALE")[SEX],]
 id <- which.max(temp$AUC_5yrs)
 select = rbind(select,temp[id,])
 }
}
select[,c("PGS.ID","simplified","SEX","AUC_5yrs")]

temp1[temp1$PGS.ID%in%c("PGS000149","PGS000055"),c("PGS.ID","AUC_5yrs","simplified","SEX")]

## Lung and colorectal cancer - combined sex

temp1. = NULL
for(CANCER in cancer.list[c(3,4)]){
 temp <- auc.table(DATA=data[data$V9_gender==SEX,],
 COL.PHENO=paste0("PHENO.",CANCER),
 COL.TIME=paste0("TIME.",CANCER),
 VAR.LIST=scores.list.[[CANCER]],
 MEAN.SD=mean.sd[mean.sd$Sex=="ALL",],
 NO.YEAR=20)
 temp$PHENO <- CANCER
 temp1. <- rbind(temp1.,temp)

}
output.auc <- temp1.

temp1. = NULL
for(CANCER in cancer.list[c(3,4)]){
 temp <- surv.auc.table(DATA=data[data$V9_gender==SEX,],
 COL.PHENO=paste0("PHENO.",CANCER),
 COL.TIME=paste0("TIME.",CANCER),
 VAR.LIST=scores.list.[[CANCER]],
 MEAN.SD=mean.sd[mean.sd$Sex=="ALL",],
 NO.YEAR=20)
 temp$PHENO <- CANCER
 temp1. <- rbind(temp1.,temp)

}
output.auc.surv <- temp1.


# distribution
temp1. = NULL
for(CANCER in cancer.list){
 temp <- dis.table(DATA=data[data$V9_gender==SEX,],
 COL.PHENO=paste0("PHENO.",CANCER),
 COL.TIME=paste0("TIME.",CANCER),
 VAR.LIST=scores.list.[[CANCER]],
 MEAN.SD=mean.sd[mean.sd$Sex=="ALL",],
 NO.YEAR=20)
 temp$PHENO <- CANCER
 temp1. <- rbind(temp1.,temp)
}
output.distribution <- temp1.


output <- merge(output.auc,output.auc.surv,by=c("PRS","PHENO"))
output <- merge(output.distribution,output,by=c("PRS","PHENO"))

select.auc = select.auc.surv = NULL
for(CANCER in cancer.list[c(3,4)]){
 temp <- output[output$PHENO==CANCER,]
 id <- which.max(temp$auc)
 select.auc = rbind(select.auc,temp[id,])
 id <- which.max(temp$AUC_5yrs)
 select.auc.surv = rbind(select.auc.surv,temp[id,])
}

output. <- output[,c("PRS","PHENO","Mean","SD","N","Ncase","Nnoncases",
 "MeanSD_case","MeanSD_noncase",
 " MeanDifference_95%CI","Pvalue_ttest",
 "AUC (95%CI)","AUC_5yrs",
 "HR (95%CI)","P (Cox HR)","P (PH assumption)")]


write.csv(output.,paste0("output/sT6_combined_sex_additional_analysis_revision.csv"),row.names = F)

## Other features associated with performance

dir.create("plot/Features")
data.a <- read.csv(paste0("output/AUC_common_cancer_scoresum_SD_labeled_20yr_revision.csv"))
pvalue.out = NULL
for(CANCER in cancer.list){
 for(SEX in sex.list[[CANCER]]){
 pvalues = NULL
 cal.data. <- read.csv(paste0("output/Calibration_",CANCER,"_",c("MALE","FEMALE")[SEX],"_5yr_revision.csv"))
 CAL = cal.data.[cal.data.$Order==0,]
 CAL$PGS.ID <- str_replace(CAL$PGS,"SCORESUM_","")
 CAL = CAL[,c("PGS.ID","cal","HL.p")]
 DATA = data.a[data.a$SEX==c("MALE","FEMALE")[SEX] & data.a$simplified==CANCER,]
 DATA = merge(CAL,DATA,by="PGS.ID")
 fit <- lm(auc~Number.of.Variants,data=DATA)
 print(paste0(CANCER," \226 ",c("male","female")[SEX]))
 print("AUC~SNP.No.")
 print(summary(fit))
 pvalues <- rbind.data.frame(pvalues,
 c("auc",
 paste0("P=",form.2(summary(fit)$coef[2,4],dp=3)),
 max(DATA$auc)))
 fit <- lm(cal~Number.of.Variants,data=DATA)
 print("CALIBRATION~SNP.No.")
 print(summary(fit))
 pvalues <- rbind.data.frame(pvalues,
 c("cal",
 paste0("P=",form.2(summary(fit)$coef[2,4],dp=3)),
 max(DATA$cal)))
 fit <- lm(HL.p~Number.of.Variants,data=DATA)
 print("Hosmer-Lemeshow P~SNP.No.")
 print(summary(fit))
 pvalues <- rbind.data.frame(pvalues,
 c("HL.p",
 paste0("P=",form.2(summary(fit)$coef[2,4],dp=3)),
 max(DATA$HL.p)))

 label.facet <- c("AUC","Calibration","Hosmer-Lemeshow P")
 names(label.facet) = c("auc","cal","HL.p")
 DATA.PLOT <- melt(DATA[,c("PGS.ID","simplified","SEX","Number.of.Variants","auc","cal","HL.p")],id.vars = c("PGS.ID","simplified","SEX","Number.of.Variants"))
 DATA.PLOT = DATA.PLOT[DATA.PLOT$Number.of.Variants<10000,]
 colnames(pvalues) <- c("variable","P","y")
 pvalues$x <- max(DATA.PLOT$Number.of.Variants)

 plot. <- ggplot(data=DATA.PLOT,aes(x=Number.of.Variants,y=value)) +
 geom_point() +
 geom_text(data=pvalues,aes(x=as.numeric(x),y=as.numeric(y),label=P),hjust=1,vjust=1) +
 facet_wrap(facets = "variable",nrow=3,scales = "free_y",labeller = labeller(variable=label.facet)) +
 labs(title=paste0(CANCER,"-",c("Male","Female")[SEX]),
 x="Number of variants")
 png(paste0("plot/Features/",CANCER,"-",c("Male","Female")[SEX],".png"),res=300,width=1500,height=1500)
 print(plot.)
 dev.off()

 pvalue.out = rbind.data.frame(pvalue.out,cbind.data.frame(CANCER,c("Male","Female")[SEX],pvalues))
 }
}

write.csv(pvalue.out,"output/features.csv",row.names = F)

# Incidence rates as compared to the Singapore’s population

Incidence rates, lower (aged 40 years) and upper (aged 74 years) bounds from the Singapore Cancer Registry 2013-2017

PY=100000
female = 12084
male = 9610
median.year = 20

breast = 495
breast/female/median.year *PY #SCHS
189.4 #Age40
255 #Age60
248.8 #Age74

prostate = 308
prostate/male/median.year *PY#SCHS
0.8 #Age40
126 #Age60
401.2 #Age74

colorectal.female = 332
colorectal.female/female/median.year *PY
8.9 #Age40
63.3 #Age60
132.6 #Age74

colorectal.male = 409
colorectal.male/male/median.year *PY
9.4 #Age40
89.5 #Age60
199.2 #Age74

lung.female = 181
lung.female/female/median.year *PY
6.7 #Age40
58.4 #Age60
112.4 #Age74

lung.male = 381
lung.male/male/median.year *PY
7.3 #Age40
110.7 #Age60
327.1 #Age74
